# CRISPR-Cas9-mediated pinpoint microbial genome editing aided by target-mismatched sgRNAs

**DOI:** 10.1101/gr.257493.119

**Published:** 2020-05

**Authors:** Ho Joung Lee, Hyun Ju Kim, Sang Jun Lee

**Affiliations:** Department of Systems Biotechnology, and Institute of Microbiomics, Chung-Ang University, Anseong 17546, South Korea

## Abstract

Genome editing has been revolutionized by the CRISPR-Cas9 system. CRISPR-Cas9 is composed of single-molecular guide RNA (sgRNA) and a proteinaceous Cas9 nuclease, which recognizes a specific target sequence and a protospacer adjacent motif (PAM) sequence and, subsequently, cleaves the targeted DNA sequence. This CRISPR-Cas9 system has been used as an efficient negative-selection tool to cleave unedited or unchanged target DNAs during site-specific mutagenesis and, consequently, obtain microbial cells with desired mutations. This study aimed to investigate the genome editing efficiency of the CRISPR-Cas9 system for in vivo oligonucleotide-directed mutagenesis in bacteria. This system successfully introduced two- to four-base mutations in *galK* in *Escherichia coli* with high editing efficiencies (81%−86%). However, single-point mutations (T504A or C578A) were rarely introduced with very low editing efficiencies (<3%), probably owing to mismatch tolerance. To resolve this issue, we designed one- or two-base mismatches in the sgRNA sequence to recognize target sequences in *galK* in *E. coli*. A single-point nucleotide mutation (T504A or C578A in the *galK* gene) was successfully introduced in 36%−95% of negatively selected *E. coli* cells using single-base mismatched sgRNAs. Sixteen targets were randomly selected through genome-wide single-base editing experiments using mismatched sgRNAs. Consequently, out of 48 desired single-base mutations, 25 single bases were successfully edited, using mismatched sgRNAs. Finally, applicable design rules for target-mismatched sgRNAs were provided for single-nucleotide editing in microbial genomes.

The function of bacterial clustered regularly interspaced short palindromic repeats (CRISPR) is to “memorize” certain DNA sequences derived from foreign genetic elements and to degrade invasive genetic materials, such as alien plasmids and bacteriophages (Barrangou and Marraffini 2014; Westra et al. 2014). It is, therefore, referred to as a bacterial adaptive immune system. Numerous studies have investigated the biological roles and ubiquitous existence of CRISPR in bacterial genomes since the discovery of specific repeated nucleotide sequences three decades ago (Ishino et al. 1987; Mojica et al. 2000, 2005; Barrangou et al. 2007). The CRISPR/Cas system is composed of functionally modular sgRNAs and Cas proteins, which can recognize target nucleotide sequences and degrade target DNAs or RNAs. The CRISPR-Cas9 system derived from *Streptococcus pyogenes*, selected from among diverse CRISPR systems, is commonly used as a genome editing tool because it contains a simple complex composed of a single Cas9 polypeptide and sgRNA, which causes the double-strand breakage (DSB) of target DNAs (Makarova et al. 2015, 2018; Le Rhun et al. 2019).

Target recognition by CRISPR-Cas9 is not entirely determined by DNA–RNA hybridization between the single-stranded target DNA and target-recognizing sgRNA. The presence of protospacer adjacent motifs (PAMs) of 5′-NGG immediately after the target DNA (N_1_–N_20_) is important for the CRISPR-Cas9 system to distinguish self- and nonself DNA (Sternberg and Doudna 2015; Moon et al. 2019). The interaction between the 5′-NGG PAM sequence and several amino acid residues of the Cas9 protein is critical for cleaving double-stranded target DNAs by Cas9–sgRNA complexes (Sander and Joung 2014). Because the complexity of PAM sequences may limit the availability of targetable genomic sequences (Hsu et al. 2014), other systems with alternative PAM sequences, such as CRISPR-Cpf1, have been developed and used for genome editing (Koonin et al. 2017).

The CRISPR-Cas9 system was first used to introduce precise mutations in the genomes of *Streptococcus pneumoniae* and *Escherichia coli* in combination with lambda Red recombineering (Jiang et al. 2013). When mutations are introduced into the bacterial genome via oligonucleotide-directed mutagenesis, unedited cells are expected to be eliminated by DSBs at unchanged targets by CRISPR-Cas9, and only the edited cells are expected to survive; this is called negative selection. Thus far, various CRISPR-based genome editing technologies have been developed to induce mutations, including substitution and indels across diverse bacterial species for basic genetic studies and biotechnological applications (Vento et al. 2019).

A previous study introduced a 6-bp mismatch and a 3-bp mutation in the *E. coli* genome with high efficiencies of 50%−80% (Pyne et al. 2015) and 94%−99% (Reisch and Prather 2015), respectively, through lambda Red–mediated recombineering of oligonucleotides, followed by CRISPR-Cas9 negative selection. Because the lambda Bet protein promotes recombineering of single-stranded DNA (Costantino and Court 2003), Bet-mediated recombineering along with CRISPR-Cas9 facilitated genomic editing of two to three bases in *galK*, *xylA*, and *lacZ* in *E. coli* with very high efficiencies (96.5%−99.7%) (Ronda et al. 2016). However, single-base editing is uncommon, probably owing to the mismatch tolerance of CRISPR-Cas9, which might recognize the single-base edited sequence as the target.

In this study, to obtain single-base edited cells, we designed one or two mismatched sequences in the target recognition sequences of sgRNAs. To determine whether negative selection using the mismatched sgRNAs is effective, various single-point mutations creating stop codons in *galK* were tested as the target of CRISPR-Cas9 in *E. coli*. Furthermore, the number of mismatched base pairs in the PAM distal or proximal regions was assessed. Moreover, genome-wide single-base editing experiments were performed to clarify design rules for mismatched sgRNAs for successful negative selection.

## Results

### Generation of multiple-base mutations through CRISPR-Cas9 negative selection

**Figure 1.**
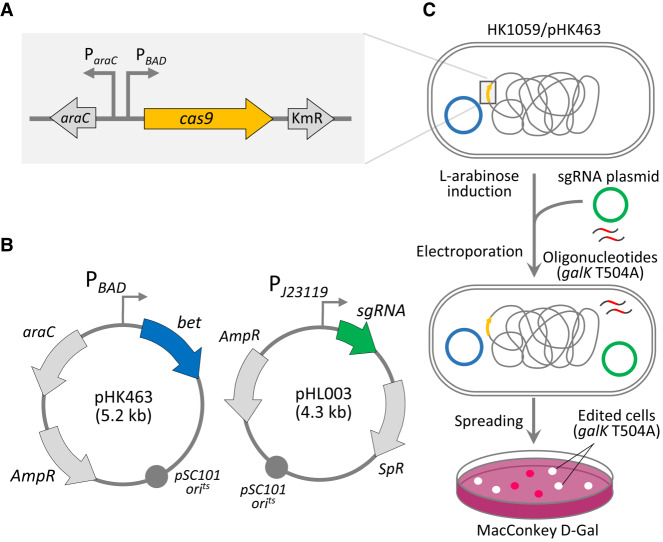
Schematic representation of bacterial genome-editing. (*A*) Chromosomal construction of *cas9* downstream from the P*_BAD_* promoter, which forms an L-arabinose-inducible *cas9* gene. (*B*) Construction of plasmids. Lambda *bet* expression vector (pHK463) was derived from pKD46 plasmid. The sgRNA expression plasmid (pHL003) harbors a temperature-sensitive origin for iterative genome engineering. (*C*) Genome editing. Mutagenic oligonucleotides carrying *galK* T504A and sgRNA plasmids were electroporated into *E. coli* cells overexpressing Cas9 and Bet proteins by L-arabinose induction. Recovered cells were spread on MacConkey agar containing D-galactose. Red/white colonies were counted for determination of *galK* editing efficiency.

Mutagenic oligonucleotides introducing stop codons (C168Z) in *galK* and sgRNA plasmids were electroporated into both Cas9 nuclease-overexpressing and lambda Bet protein–overexpressing *E. coli* MG1655 cells. If *galK* mutations were generated as intended, white colonies with the corresponding Gal^−^ phenotype would be observed on MacConkey agar plates owing to premature translation termination of the nascent galactokinase protein (Fig. 1). The white and red colonies were enumerated to determine the genome editing efficiency. Cas9/sgRNA complexes can recognize the target (N_20_) sequence (^498^AGGCTGTAACTGCGGGATCA^517^ in the *galK* gene) and generate DNA DSBs in the *galK* target, ultimately eliminating the unedited cells.

**Figure 2.**
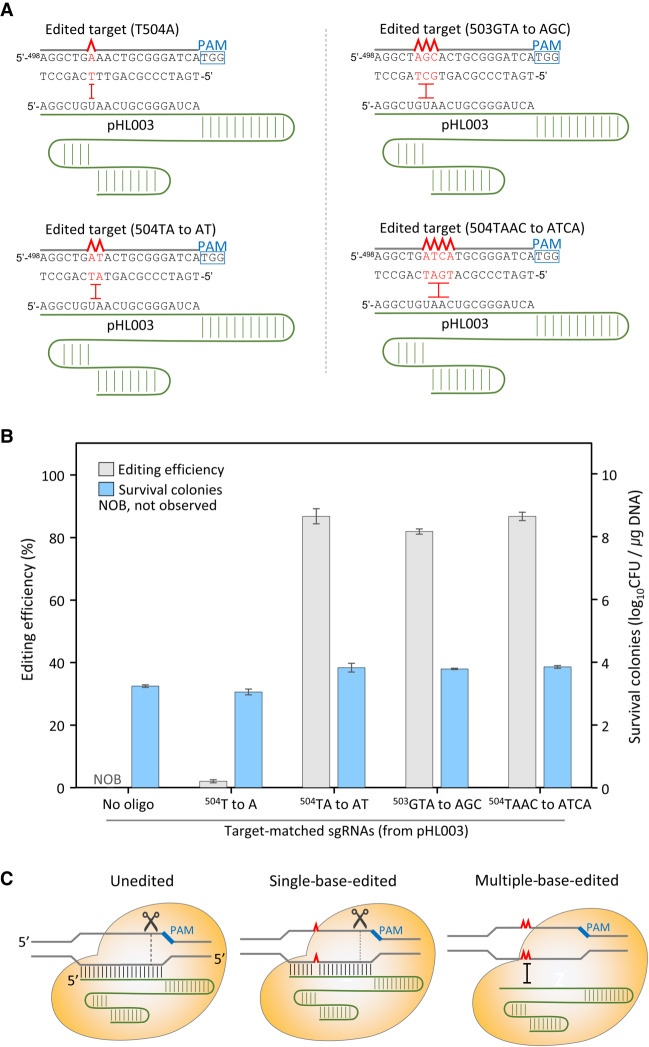
Genome editing efficiency of oligonucleotide-directed mutagenesis followed by Cas9/sgRNA-mediated negative selection. (*A*) Design of target-matched sgRNAs for negative selection of edited targets in *galK*. (*B*) The editing efficiency of the *galK* mutation using single-, double-, triple-, and quadruple-base mutagenic oligonucleotides. Each bar represents the mean of three independent experiments. (*C*) Cleavage of both unedited and single-base-edited targets by CRISPR-Cas9 owing to mismatch tolerance.

Consequently, two- to four-base substitutions (^504^TA to AT, ^503^GTA to AGC, and ^504^TAAC to ATCA) were successfully introduced, with editing efficiencies of 81%–86% (Fig. 2). However, single-point mutations (^504^T to A) were rarely introduced with very low editing efficiencies (2%) in *galK*. These results indicate that even single-base mutated DNA targets may be recognized as unchanged DNA targets and digested by the Cas9/sgRNA complex in cells; this is referred to as the mismatch tolerance of the CRISPR-Cas9 system (Lin et al. 2014; Fu et al. 2016).

In the absence of mutagenic oligonucleotides, all unedited cells would be expected to be eliminated by CRISPR-Cas9. However, numerous colonies survived (>10^3^ CFU/µg), probably owing to the existence of a subpopulation (approximately one out of 10^5^ cells) of cells wherein the CRISPR-Cas9 is not functional.

### Single-base genome editing with target-mismatched sgRNAs

**Figure 3.**
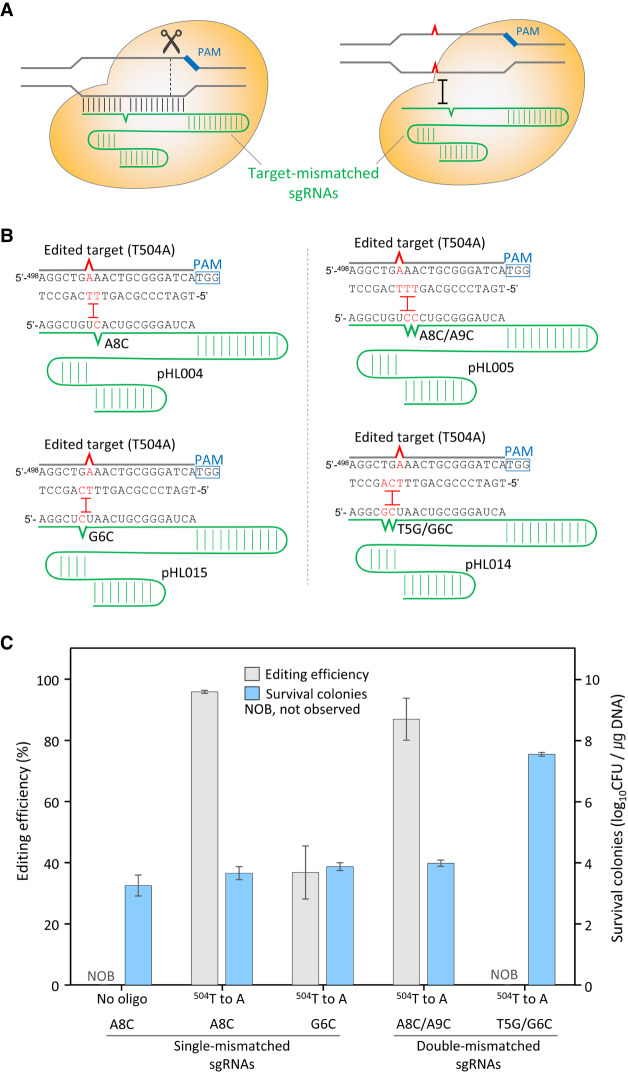
Negative selection of single-base-edited DNA targets aided by target-mismatched sgRNAs via CRISPR-Cas9. (*A*) Mismatch tolerance allows unedited targets to be cleaved by the Cas9/target–mismatched sgRNA complex. Single-base-edited targets cannot be recognized by the Cas9/target–mismatched sgRNA complex owing to multiple mismatches. (*B*) Design of target-mismatched sgRNAs for single-base editing (T504A) in *galK*. Single or double mismatch(es) were located adjacent to the T504A point mutation site. (*C*) Single-base genome editing efficiencies using single- or double-base mismatched sgRNAs and the number of surviving cells. Each bar represents the mean of three independent experiments.

Based on the successful results of multiple substitutions, we generated mismatched sgRNAs to overcome the observed mismatch tolerance (Fig. 3A). DNA targets harboring single-base substitutions were presumed to not be recognized by Cas9/target-mismatched sgRNA complexes. The sgRNA target recognition sequence was designed to harbor one or two mismatches located contiguously, flanking the point mutagenic base (^504^T) in the mutagenic oligonucleotide (Fig. 3B). When a single-point mutation is introduced correctly in the genome, two mismatches can be generated between one base-mismatched sgRNAs and single-base mutated target DNAs.

Consequently, a single-point mutation (T504A) in the *galK* gene was successfully introduced using single-base mismatched sgRNAs. When using sgRNA (A8C), the editing efficiency was 95%, determined as the number of white colonies formed by a nonsense *galK* mutation (T504A) divided by the total number of white and red colonies formed on MacConkey agar plates containing D-galactose and spectinomycin (Fig. 3C). Ten white colonies were selected, and the T504A mutation in *galK* was confirmed through Sanger DNA sequencing. In the case of sgRNA (G6C), white colonies were obtained with a decreased editing efficiency (36%). Furthermore, double-base mismatched sgRNAs (A8C/A9C and T5G/G6C) were assessed. With sgRNA (A8C/A9C), a single-point mutation (T504A) was introduced with 86% efficiency. However, when another sgRNA (T5G/G6C) was used, we observed no white colonies on the MacConkey plate, indicating unsuccessful negative selection. The survival rate for T5G/G6C was increased to 3.4 × 10^7^ CFU/μg DNA, presumably because the Cas9/sgRNA (T5G/G6C) complex cannot appropriately generate DSBs at the unchanged target DNAs in the unedited cells.

### Pinpoint genome editing by target-mismatched sgRNAs in the PAM proximal region

**Figure 4.**
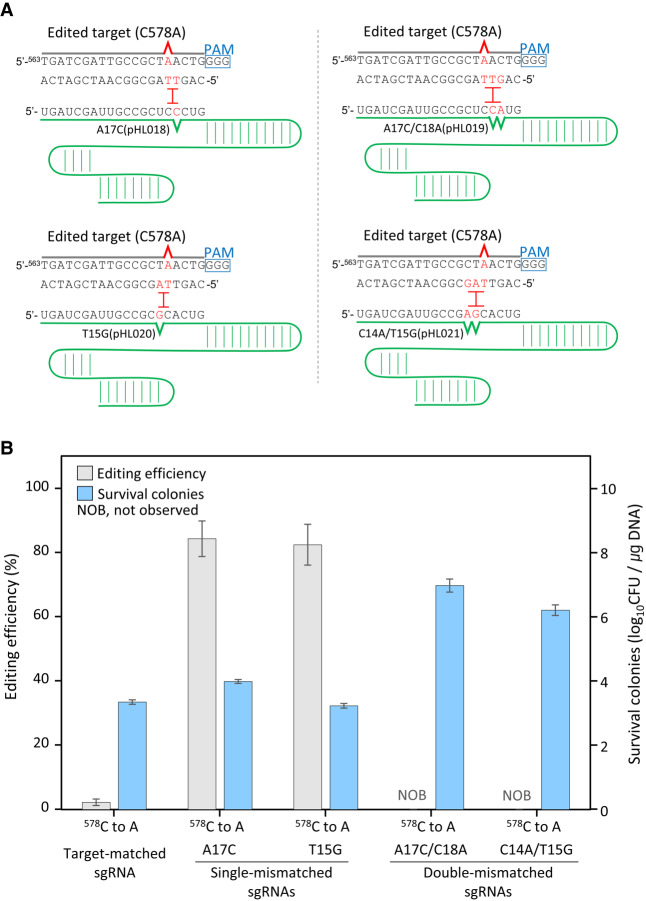
Single-base editing in the PAM-proximal region using target-mismatched sgRNAs. (*A*) Negative selection of edited target sequence (C578A in the *galK* gene) by Cas9/target-mismatched sgRNA complexes. Single or double mismatch(es) were located adjacent to the C578A point mutation site. (*B*) Genome editing efficiencies using target-matched, single- or double-base mismatched sgRNAs and the number of surviving cells. Each bar represents the mean of three independent experiments.

Base-paring between sgRNA and the target DNA in the PAM proximal region is more critical for target recognition and cleavage by CRISPR-Cas9 (Cencic et al. 2014; Anderson et al. 2015). Therefore, proximal mismatches between sgRNA and the target DNA were assessed herein (Fig. 4A). In the case of target-matched sgRNA for single-point mutation (C578A) in the *galK* gene, we obtained some white colonies (<2.3%). In the case of single-base mismatched sgRNAs (A17C and T15G), the editing efficiencies were 84% and 82%, respectively, and the number of surviving cells was <10^4^ CFU/μg DNA. However, in the case of double-mismatched sgRNAs (A17C/C18A, and C14A/T15C), no white colonies were observed and survival rates were markedly higher (≥10^6^ CFU/μg DNA), indicating that Cas9/double base-mismatched sgRNA complexes cannot recognize the unedited targets (Fig. 4B). These results show that single-base mismatched sgRNAs allow efficient negative selection, regardless of mismatches in the PAM proximal or distal region.

### Plasmid curing and deletion of *cas9* for scar-free genome engineering

**Figure 5.**
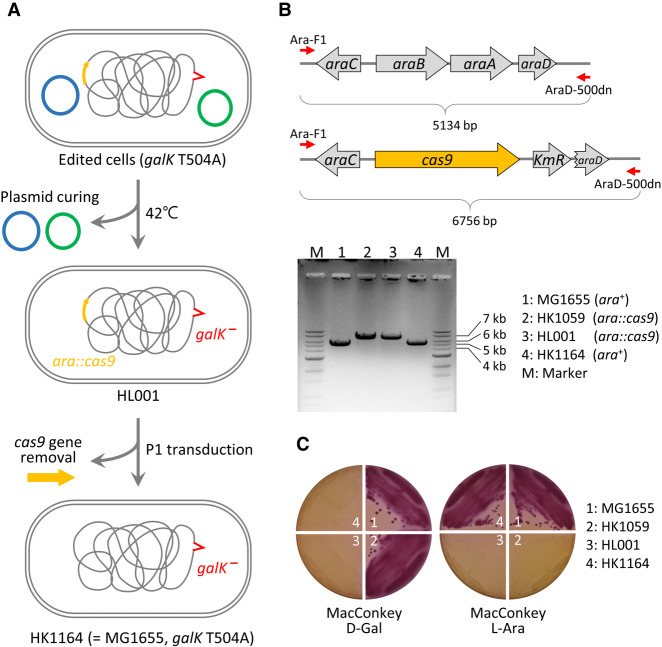
Scar-free single-base genome engineering. (*A*) Plasmids in the edited cells were cured through incubation at a high temperature, and the *cas9*-KmR cassette was replaced with the *araBAD* operon through P1 transduction. (*B*) The chromosomal structures in the *ara* operon in *E. coli* MG1655, HK1059, HL001, and HK1164 cells were confirmed via PCR. (*C*) The phenotype (Gal^−^Ara^+^) of single-base-edited HK1164 cells (=MG1655, *galK* T504A), as confirmed through cell culture on MacConkey agar supplemented with D-galactose or L-arabinose.

All sgRNA plasmids harbor a temperature-sensitive origin (pSC101 *ts-ori*) that can be cured through incubation at 42°C, enabling iterative genome editing using different oligonucleotides and sgRNA plasmids. After genome editing, genome-integrated *cas9* was replaced with the original *araBAD* gene through P1 transduction, and the transformed cells were selected in L-arabinose minimal medium and confirmed through selection in MacConkey agar containing D-galactose or L-arabinose (Fig. 5). Consequently, scar-free single-base substitutions could be achieved in bacterial genomes.

### Genome-wide single-base editing using single mismatched sgRNAs

**Figure 6.**
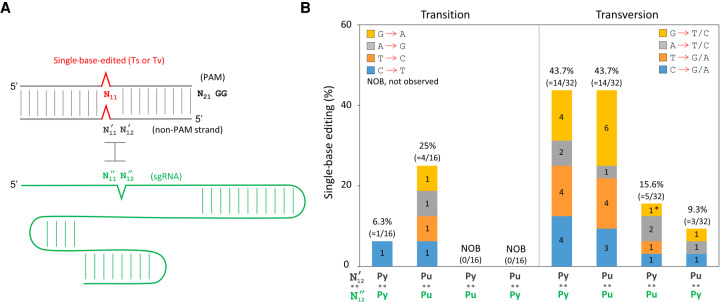
Genome-wide single-base editing aided by target-mismatched sgRNAs in CRISPR-Cas9. (*A*) Design of mismatched sgRNAs for single-base-edited targets. Single mismatches (N′_12_ : N′′_12_) between non-PAM strands and sgRNAs were introduced immediately after the single-base-edited sites (N_11_). Note that mismatches in two *galK* ([504] and [578]) targets were designed at N′_8_ : N′′_8_ and N′_17_ : N′′_17_, respectively. (*B*) Single-base genome editing for 16 different DNA targets was aided by various base-parings (N′_12_ : N′′_12_). Numbers in parentheses and colored boxes are the frequency and the successful events of single-base editing, respectively. Asterisk indicates one single-base editing was observed using a target-matched sgRNA among 16 target-matched sgRNAs.

To generate applicable design rules for mismatched sgRNAs, 16 target sequences for CRISPR-Cas9 negative selection were randomly selected in the genome of *E. coli*. Base-editing sites were designed at N_11_ in target sequences, except N_7_ in *galK* (504) and N_16_ in *galK* (578), in 48 mutagenic oligonucleotides (three oligonucleotides per target, i.e., A → G/T/C, T → G/A/C, G → A/T/C, and C → G/A/T) (Supplemental Table S3). Because single-base mismatched sgRNAs accurately edited *galK* (Figs. 3, 4), three sgRNAs with different single-base mismatches (immediately adjacent to edited bases, i.e., N′_12_ : N′′_12_) in each target were used for negative selection (Fig. 6A; Supplemental Fig. S1). As a control, target-matched sgRNAs were used for each target (Supplemental Table S5). Therefore, 192 combinatorial electroporations (16 targets × 3 mutagenic oligonucleotides × 4 sgRNAs/target) were performed, and subsequent base editing in three randomly selected colonies (per target) was analyzed via PCR followed by Sanger sequencing (Supplemental Fig. S2). One, two, or three edited sequences from the three reactions (in each electroporation) were considered successful base editing.

Consequently, of 48 types of single-base editing in 16 targets (equivalent to the number of mutagenic oligonucleotides), 25 types of base changes in 13 targets were successfully obtained using mismatched sgRNAs (52% = 25/48) (Supplemental Table S6). To determine which mutations are effectively introduced by which mismatched sgRNAs, we analyzed 41 individual single-base editings (among 192 electroporated samples) for the type of base editings and mismatch patterns between DNA targets and sgRNAs.

First, we analyzed the data by base-editing types (64 transitions and 128 transversions). Among 41 single-base editings, transition changes were rarely observed (8%; 5/64). However, transversion base editings were more frequent (28%; 36/128). Among 41 cases, only one was obtained through target-matched sgRNAs. Transition at N_11_ resulted in moderate base mispairing, including Pu:Py or Py:Pu between N′_11_ in a non-PAM strand and N′′_11_ in an sgRNA; however, transversion at N_11_ caused base mispairing such as Pu:Pu or Py:Py at N′_11_ : N′′_11_.

Because base editing is apparently affected by mismatch patterns between N′_11_ and N′′_11_, 41 base-editing cases were analyzed on the basis of mispairing patterns between N′_12_ and N′′_12_ (Fig. 6B). In cases of transversions, 43.7% of single-base editings were obtained with Py:Py and Pu:Pu mispairings at N′_12_ : N′′_12_. Less efficient base editings were observed in Py:Pu (15.6%) and Pu:Py (9.3%), wherein target-matched sgRNAs were used in half of the electroporation samples (32/64). In cases of transition, no base editing was observed with Py:Pu and Pu:Py at N′_12_ : N′′_12_. However, Py:Py and Pu:Pu mismatches facilitated single-base editing with 6.3% and 25% efficiencies, respectively. These data indicate that two consecutive mismatches of Py:Py or Pu:Pu between non-PAM strands and sgRNAs appear efficient in single-base editing aided by target-mismatched sgRNAs in CRISPR-Cas9.

## Discussion

As the CRISPR-Cas9 technology has accelerated and simplified accurate genetic engineering in cells, genomic mutations occurring either spontaneously during replication or generated in vitro can be rectified. An important goal of genome editing in biotechnology applications is to introduce desired mutations into new backgrounds for directed genetic engineering of useful phenotypes (Bailey et al. 1996).

DNA DSBs caused by CRISPR-Cas9 can be restored through nonhomologous end joining (NHEJ), which can inactivate target genes through indel or frame-shift mutations. If homologous regions flanking double-stranded DNA or oligonucleotides are concomitantly added, cells harboring unchanged target sequences can be eliminated via CRISPR-Cas9, and edited cells can survive because of the lack of a target site (Mougiakos et al. 2016; Ronda et al. 2016).

Oligonucleotide-directed mutagenesis combined with CRISPR-Cas9 has been developed for convenient editing of bacterial genomes (Jiang et al. 2013; Pyne et al. 2015). However, the introduction of single-point mutations into bacterial genomes by CRISPR-Cas9 has been only rarely observed. Our experiments have shown that single-point genomic mutations cannot be obtained through negative selection using CRISPR-Cas9 (Fig. 2). Four different mutagenic oligonucleotides were electroporated into *E. coli* cells with the same sgRNA plasmid. Two- to four-base substitutions were obtained with high efficiency (81%–86%). However, no single-base mutated cell was obtained among several hundred surviving cells. This may have resulted because single-point mutations seem to be recognized as the DNA targets owing to mismatch tolerance of CRISPR-Cas9 (Jinek et al. 2012; Liang et al. 2017).

The CRISPR-Cas9 system originally evolved as a bacterial adaptive immune system. CRISPR-Cas9 has been considered to degrade external nonself DNAs from phages or mobile DNAs, notwithstanding single mutations in the target DNA or target-specific sgRNA sequences. Because the number of redundant permutations (∼4^20^) of possible target sequences, other than the PAM sequence, is markedly greater than the size of bacterial genomes (∼4^11^), one or two mismatches are still safe and tolerant to distinguish self and nonself DNA. Thus, mismatch tolerance involving CRISPR-Cas9 is inevitable and a natural consequence of evolution.

Based on our results indicating the lack of single-base mutations and because multiple-base substitution mutations are successfully obtained with high efficiency, we designed and introduced target-mismatched sequences in sgRNAs in advance so as not to recognize single-base mutated sequences as the target (Fig. 3A). Because oligonucleotide-directed mutagenesis alters at least two successive bases (Fig. 2A), one or two mismatched sequences in sgRNAs were required on both sides of a single-base substitution. Single-base mismatched sgRNAs helped us induce single-point mutations (Fig. 3C). Our results indicate that single-base mismatched sgRNAs facilitate efficient negative selection, regardless of mismatches in the PAM-proximal or PAM-distal regions (Figs. 3, 4). Moreover, among four different double-base mismatched sgRNAs (A8C/A9C, T5G/G6C, A17C/C18A, and C14A/T15G), only sgRNA (A8C/A9C) facilitated negative selection of single-point mutations, indicating that accurate negative selection cannot be expected using double-base mismatched sgRNAs.

During genome-wide single-base editing, some genes are more susceptible to base editing than others. We observed high single-base editing efficiencies (four or more of nine) with mismatched sgRNAs in four targets: *mnmE*, *fau*, *proX*, and *ydcO*. However, no single-base editing was observed in three DNA targets: *galK* (934), *ypdA*, and *yjhF* genes (Supplemental Table S6). In *galK*, several edited bases were obtained in *galK* (504) and (578) targets but not in *galK* (934), indicating that the efficiency of base editing may have been affected by target DNA sequences rather than the chromosomal location.

In the single-base editing experiments, transversion base changes were easily obtained, causing Py:Py or Pu:Pu at N′_11_ : N′′_11_ between non-PAM strands and sgRNAs, when adjacent Py:Py or Pu:Pu at N′_12_ : N′′_12_ was designed in mismatched sgRNAs for negative selection (Fig. 6). Two consecutive mismatches potentially result in inaccurate conformations of DNA (non-PAM strand) and RNA (sgRNA) duplexes, potentially misleading target recognition. Even one mismatch (Py:Py or Pu:Pu) could be better recognized as targets by CRISPR-Cas9. For G:U wobble base-pairing (Varani and McClain 2000), only one single-base editing was successful in 12 cases (Supplemental Table S6). Consequently, G:U base-pairing was not effective for negative selection. Although the sgRNA plasmid was successfully constructed, negative selection failed in six electroporation samples, wherein more than 10^7^ survival colonies (CFU/µg) were obtained.

Considering the design of target-mismatched sgRNAs for single-base editing of microbial genomes, it is important to consider the following points: (1) Transversion editing is more effective, (2) there is Py:Py or Pu:Pu base mispairing between non-PAM strands and sgRNAs, and (3) the G:U base pair is not markedly effective.

In summary, a single-base microbial genome editing method was developed from the use of mismatch tolerance, which has been an obstacle in the negative selection of unedited targets by CRISPR-Cas9 system. Target-mismatched sgRNA methods can be used for accurate genome reprogramming, including the fine-tuning of promoter strength and editing of codons of interest in microbial cells.

## Methods

### Strains and culture conditions

*E. coli* strains used herein are listed in Supplemental Table S1 and were grown in Luria-Bertani broth (LB) at 30°C or 37°C, depending on the plasmid *ori* sequence. *E. coli* DH5α and MG1655 were used as cloning hosts and for genomic integration of *cas9*. *E. coli* MG1655 cells harboring plasmid pKD46 were grown in LB containing ampicillin (50 µg/mL) at 30°C, and L-arabinose (final 1 mM) was added when the optical density at 600 nm (OD_600_) approached 0.4 to up-regulate lambda recombinases. Subsequently, electrocompetent cells were harvested, washed, resuspended in 10% glycerol solution, and stored at −80°C until electroporation for recombineering. The construction of an *E. coli* strain carrying the *cas9* gene in the chromosome is described below. When needed, kanamycin and spectinomycin were added to the culture medium at 25 and 75 µg/mL, respectively.

### Genomic integration

Primers used to construct *E. coli* HK1059 are listed in Supplemental Table S2. *cas9* was PCR-amplified using the plasmid pCas (a gift from Sheng Yang; Addgene plasmid 62225) as a template and was fused with a kanamycin-resistance marker through splice-overlap PCR to generate a *cas9*-KmR cassette. The *cas9*-KmR cassette was amplified with primer pairs harboring homologous DNA sequences for recombineering, and subsequently, the purified PCR products were electroporated into L-arabinose-induced *E. coli* MG1655 harboring plasmid pKD46 for genomic integration of the *cas9* gene in the arabinose operon. Finally, *cas9* was located downstream from the L-arabinose-inducible P_*BAD*_ promoter on the chromosome of MG1655. The strain was designated as *E. coli* HK1059.

### Plasmid construction

Plasmids used for base editing of *galK* are listed in Supplemental Table S1. The *bet* gene, and the pKD46 backbone excluding three lambda recombinase genes *exo*, *bet*, and *gam*, were amplified via PCR. These two fragments were assembled by isothermal assembly using a Gibson assembly master mix (NEB) to generate plasmid pHK463, which could only express Bet proteins after L-arabinose induction.

sgRNA expression plasmid vectors were constructed as follows. The *galK* in *E. coli* was selected as a target gene for editing. We amplified DNA fragments containing an ampicillin-resistance gene and the temperature-sensitive origin of replication in the pKD46 plasmid. The spectinomycin-resistance gene and the sgRNA gene were amplified using pTargetF (a gift from Sheng Yang; Addgene plasmid 62226) as a template. Two fragments were digested with ClaI and NcoI endonucleases and ligated to each other to form pHL001. Other sgRNA plasmids (Supplemental Table S1) were generated through Gibson assembly, using pHL001 as a template.

### Base editing in *galK*

*E. coli* HK1059 harboring pHK463 was cultured in LB supplemented with ampicillin (50 µg/mL) at 30°C, and L-arabinose (final 1 mM) was added at an OD_600_ of 0.4 to up-regulate Cas9 nucleases and lambda Bet proteins for oligonucleotide-directed mutagenesis. Thereafter, the cells were prepared and stored at −80°C for subsequent electroporation of mutagenic oligonucleotides and sgRNA plasmids. Mutagenic oligonucleotides (Supplemental Table S2) were designed to introduce a multiple-base substitution (TA504AT, GTA503AGC, or TAAC504ATCA) or single-base substitution (T504A or C578A), each introducing a premature stop codon in *galK*. For negative selection of mutant cells harboring single-base substitutions, mismatched sgRNA plasmids were designed to harbor one or two mismatches with the target (N_20_). The mismatches were located adjacent to the point mutation sites (Figs. 3B, 4A).

Each mutagenic oligonucleotide (100 pmol), with sgRNA plasmids (200 ng), was electroporated in *E. coli* HK1059 cells harboring pHK463 to introduce one- to four-base substitutions and for CRISPR-Cas9-mediated negative selection. Electroporation was performed at 25 µF, 200 Ω, and 1.8 kV, and a 0.1-cm electroporation cuvette was used. Immediately thereafter, the cells were transferred to 950 mL of SOC and incubated at 30°C, at 180 rpm for 1 h for recovery, and then spread on MacConkey agar containing D-galactose (0.5%) and spectinomycin (75 µg/mL) and incubated for 24 h at 30°C. The genome editing efficiencies of oligo-directed mutagenesis of *galK* followed by CRISPR-Cas9 negative selection were calculated by counting white and red colonies expressing D-galactose-fermenting phenotypes (Costantino and Court 2003). Surviving cells were enumerated to confirm whether target digestion by the CRISPR-Cas9 system was efficient.

### Elimination of the editing tool

After editing, *cas9* nuclease was replaced with the original *araBAD* operon through P1 transduction. To transfer the *araB*, *araA*, and *araD* genes of the MG1655 strain to an HK1059 background, we used P1 lysates of MG1655 to transduce HK1059 recipient cells (Fig. 5A). Subsequently, transduced cells were selected on M9 minimal medium containing 0.4% L-arabinose. Gene replacement by P1 transduction was confirmed via PCR (Fig. 5B).

### Genome-wide single-base editing

Sixteen targets including three targets in *galK* were randomly selected in the *E. coli* genome, and single-base mutagenic oligonucleotides for 16 targets are listed in Supplemental Table S3. sgRNA plasmids for these 16 targets were constructed through Gibson assembly, using pTargetF harboring pBR322 *ori* as a template and primers listed in Supplemental Table S4. Finally, plasmids expressing target-matched sgRNAs and single mismatched sgRNAs are listed in Supplemental Table S5.

For genome-wide single-base editing experiments, each single-base mutagenic oligonucleotide (100 pmol) and corresponding sgRNA plasmid (200 ng) were electroporated into L-arabinose-induced HK1059 cells harboring pHK463. Electroporated samples were spread on LB agar plates containing spectinomycin (75 µg/mL). After 20 h at 37°C, three colonies (per electroporation) were randomly selected, and Sanger sequencing was performed to confirm the desired base editing in the bacterial genome using primers listed Supplemental Table S4.

## Competing interest statement

H.J.L., H.J.K., and S.J.L. have filed a patent application based on this work.

## Supplementary Material

Supplemental Material
