# Supplementary material for: CRISPR-Cas9-mediated pinpoint microbial genome editing aided by target-mismatched sgRNAs
Source: Genome Res. 2020 May;30(5):768–75. doi: 10.1101/gr.257493.119 (PMC7263196; doi:10.1101/gr.257493.119)
Supplement: Supplemental Material [file supp_30_5_768__index.html]

CRISPR-Cas9-mediated pinpoint microbial genome editing aided by target-mismatched sgRNAs — CRISPR-Cas9-mediated pinpoint microbial genome editing aided by target-mismatched sgRNAs — Supplemental Material 

# CRISPR-Cas9-mediated pinpoint microbial genome editing aided by target-mismatched sgRNAs

## Supplemental Material

- Supplemental\_Fig\_S1.pdf
- Supplemental\_Fig\_S2.pdf
- Supplemental\_Table\_S1.xlsx
- Supplemental\_Table\_S2.xlsx
- Supplemental\_Table\_S3.xlsx
- Supplemental\_Table\_S4.xlsx
- Supplemental\_Table\_S5.xlsx
- Supplemental\_Table\_S6.xlsx
