# Supplementary material for: CRISPR-Cas9-mediated pinpoint microbial genome editing aided by target-mismatched sgRNAs
Source: Genome Res. 2020 May;30(5):768–75. doi: 10.1101/gr.257493.119 (PMC7263196; doi:10.1101/gr.257493.119)
Supplement: Supplemental Material [file supp_gr.257493.119_Supplemental_Fig_S1.pdf]

Figure S1.

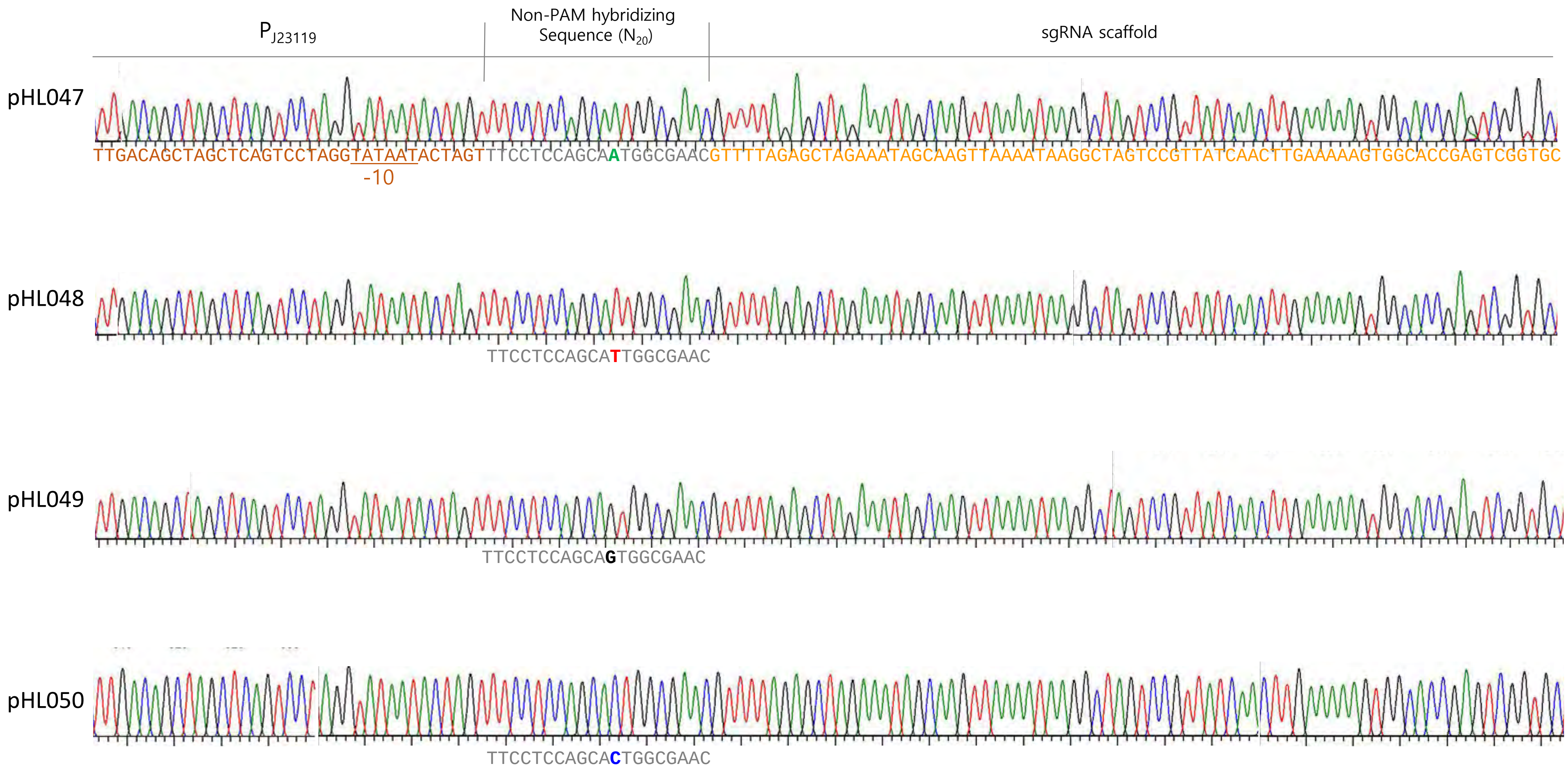

P_J23119
Non-PAM hybridizing Sequence (N20)
sgRNA scaffold
pHL047
TTGACAGCTAGCTCAGTCCTAGGTATAATACTAGTTTCCTCCAGCAATGGCGAACGTTTTAGAGCTAGAAATAGCAAGTTAAAATAAGGCTAGTCCGTTATCAACTTGAAAAAGTGGCACCGAGTCGGTGC
-10
pHL048
TTCCTCCAGCATTGGCGAAC
pHL049
TTCCTCCAGCAGTGGCGAAC
pHL050
TTCCTCCAGCACTGGCGAAC

Figure S1. (continued)

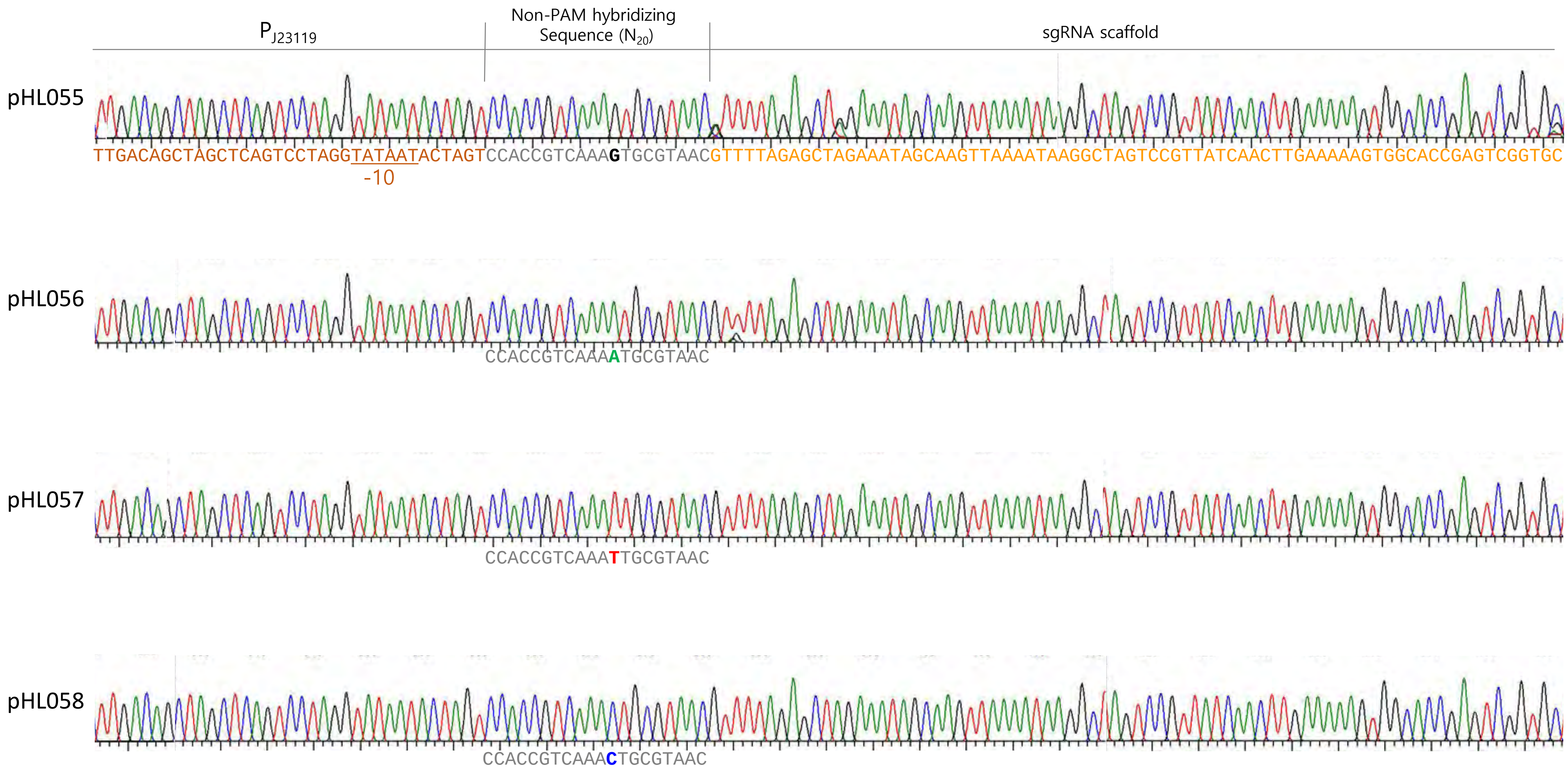

Figure S1. (continued)

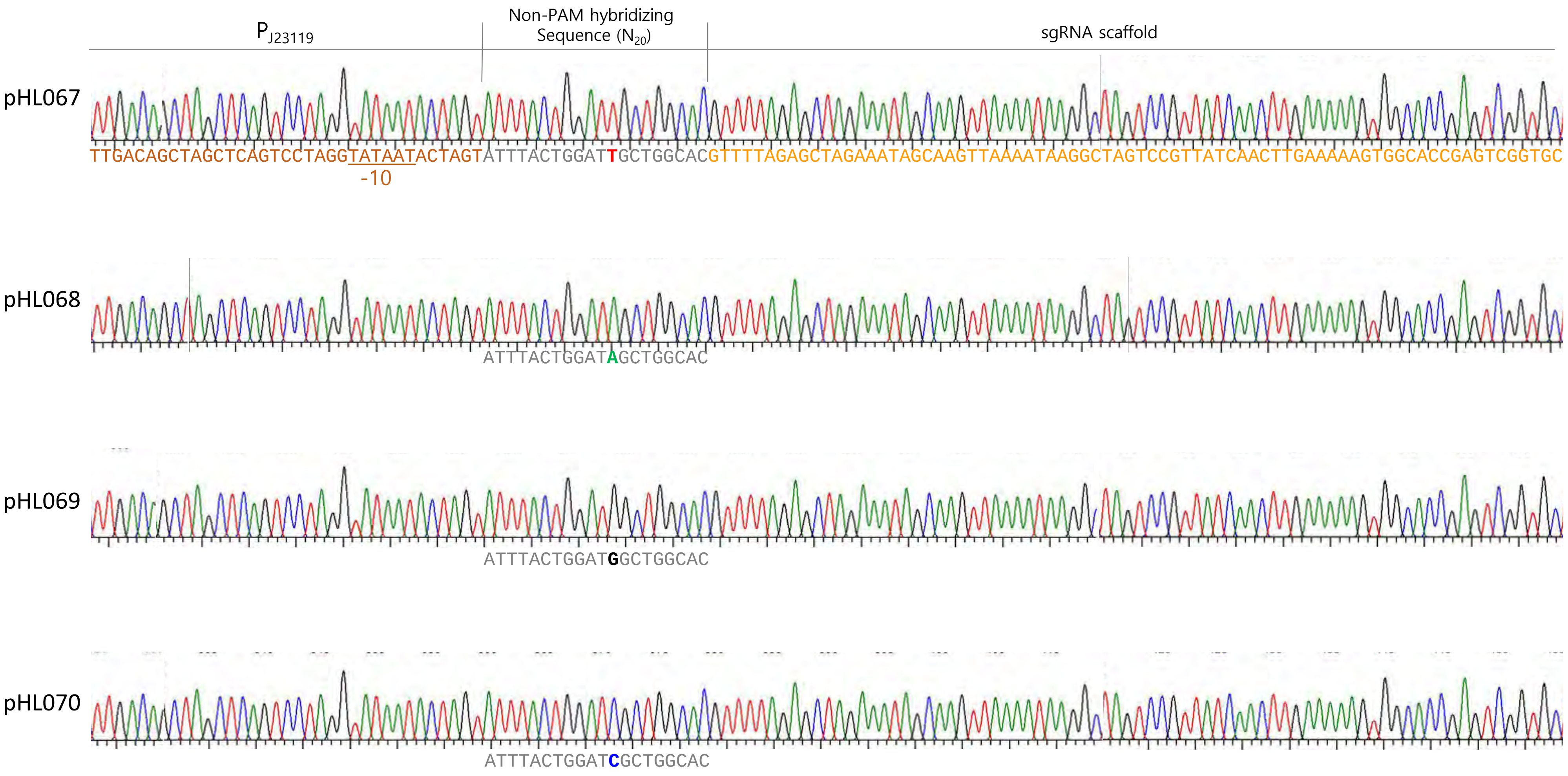

Figure S1. (continued)

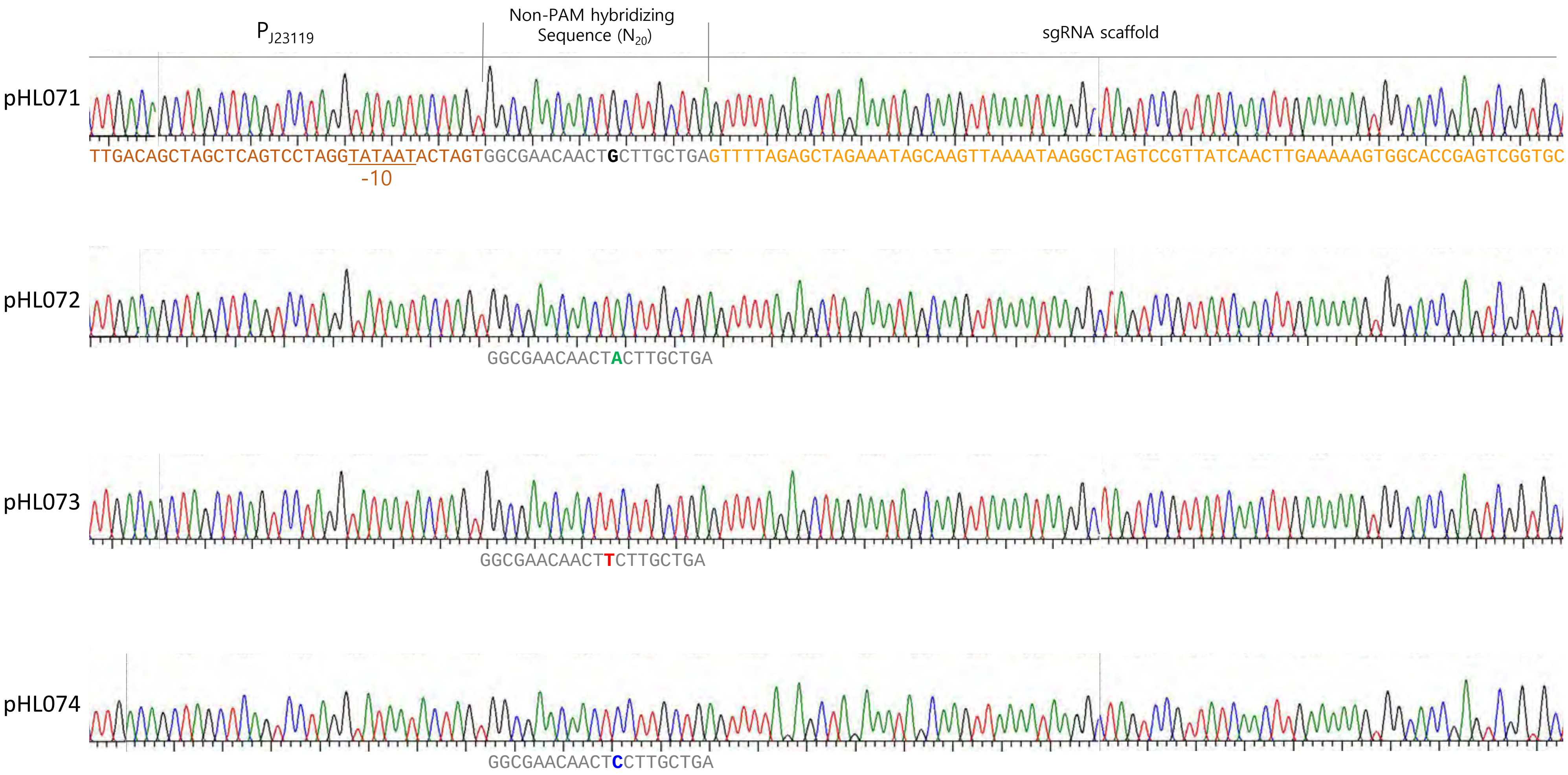

Figure S1. (continued)

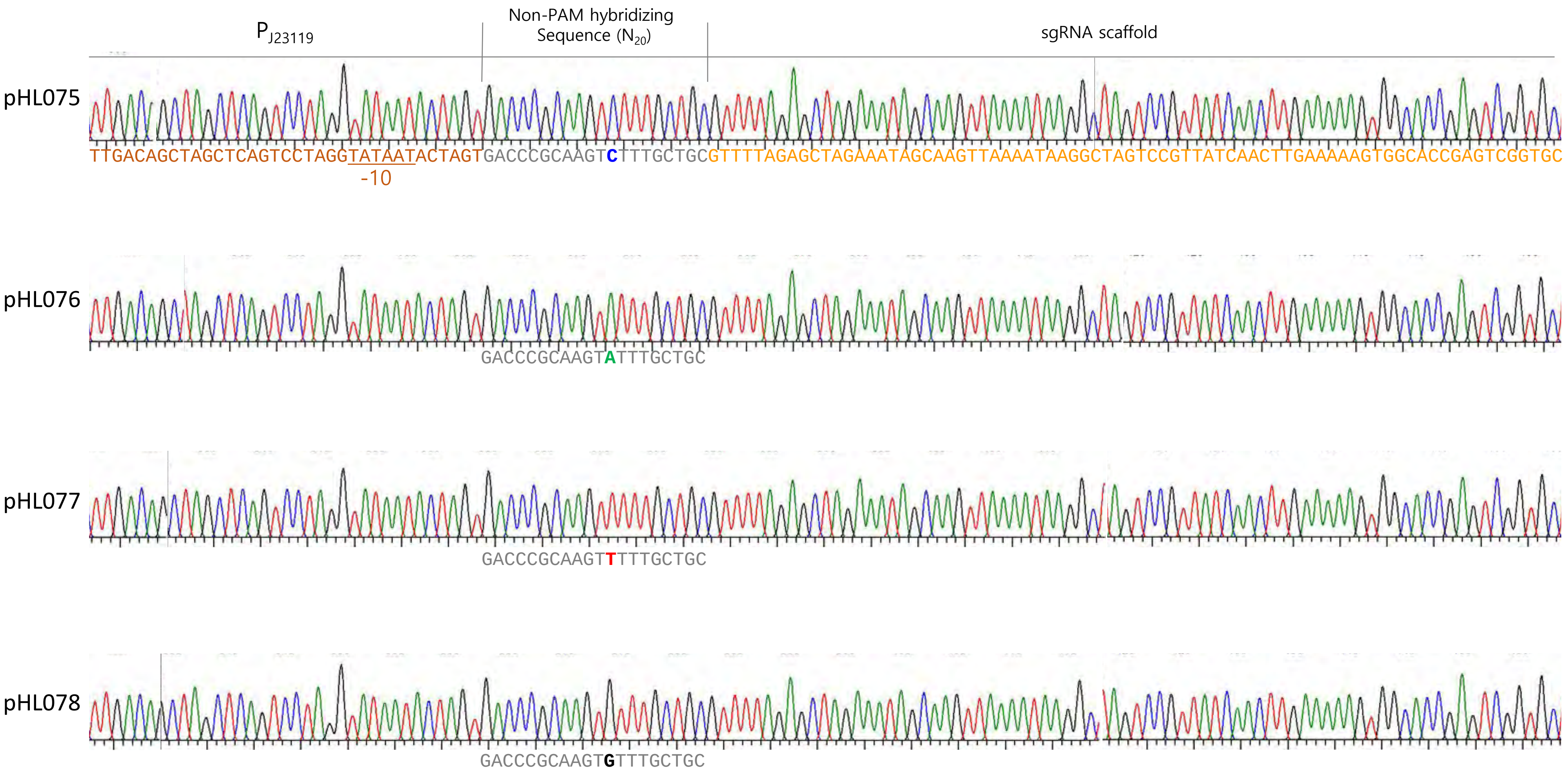

Figure S1. (continued)

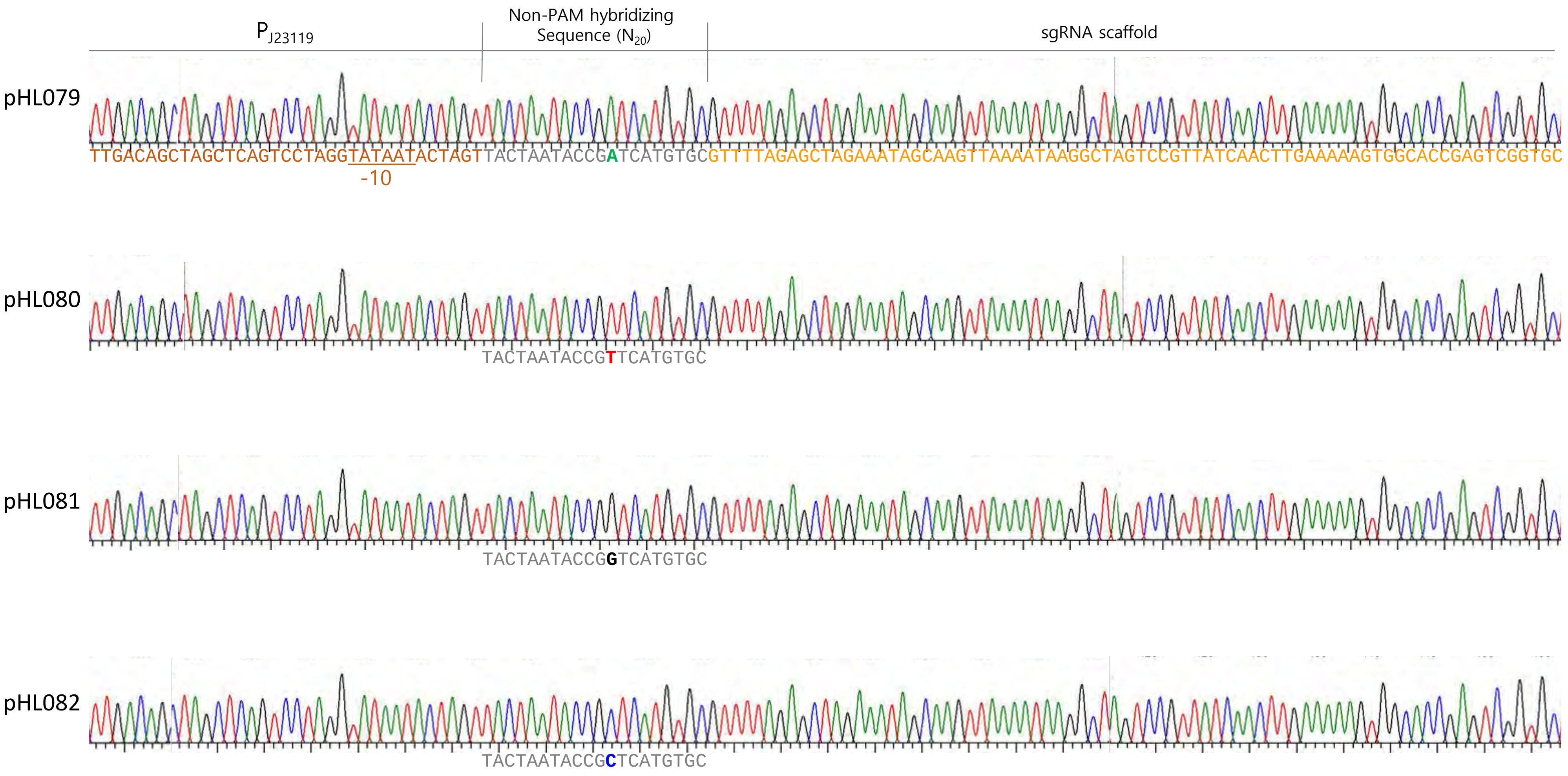

Figure S1. (continued)

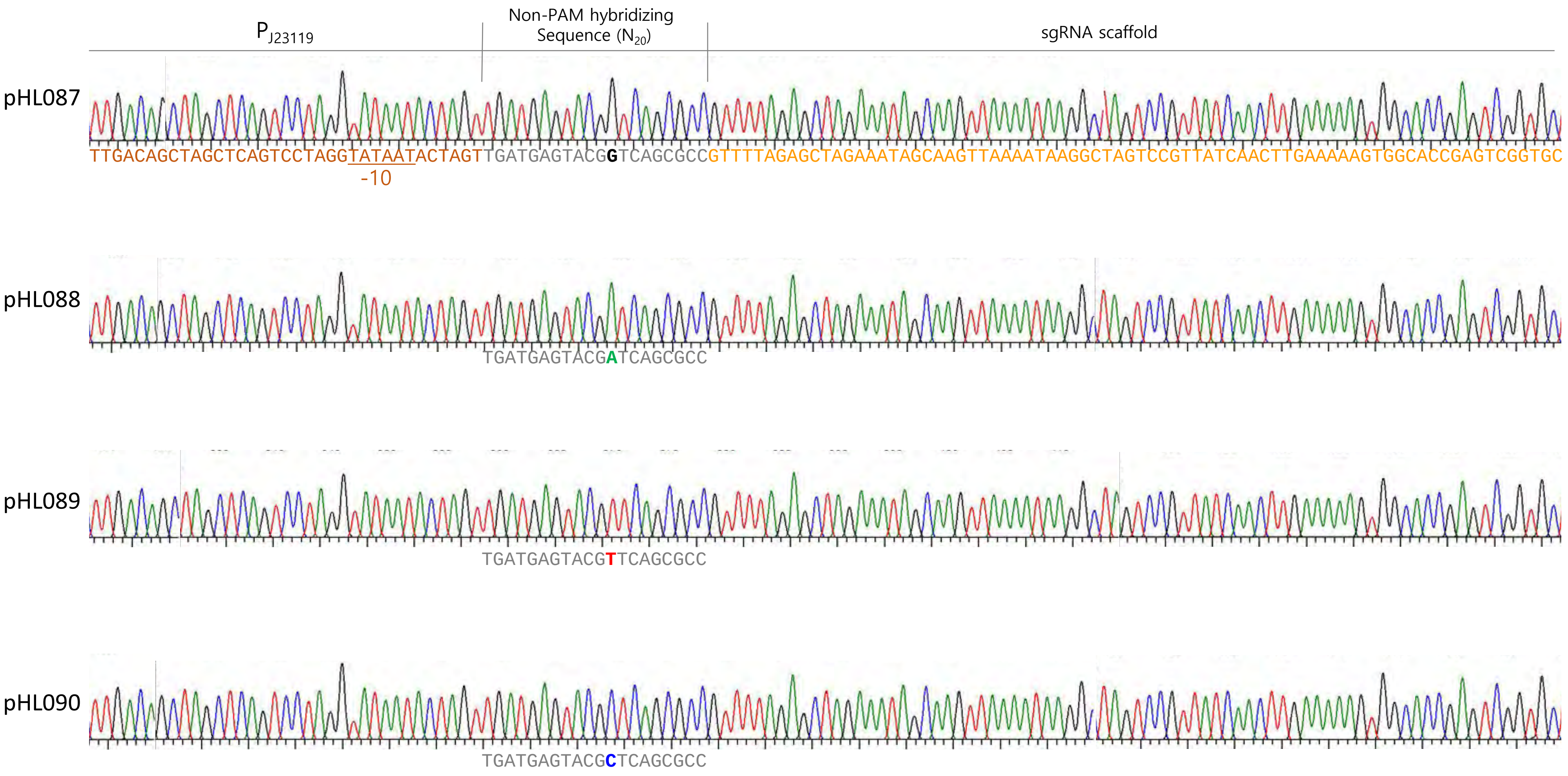

Figure S1. (continued)

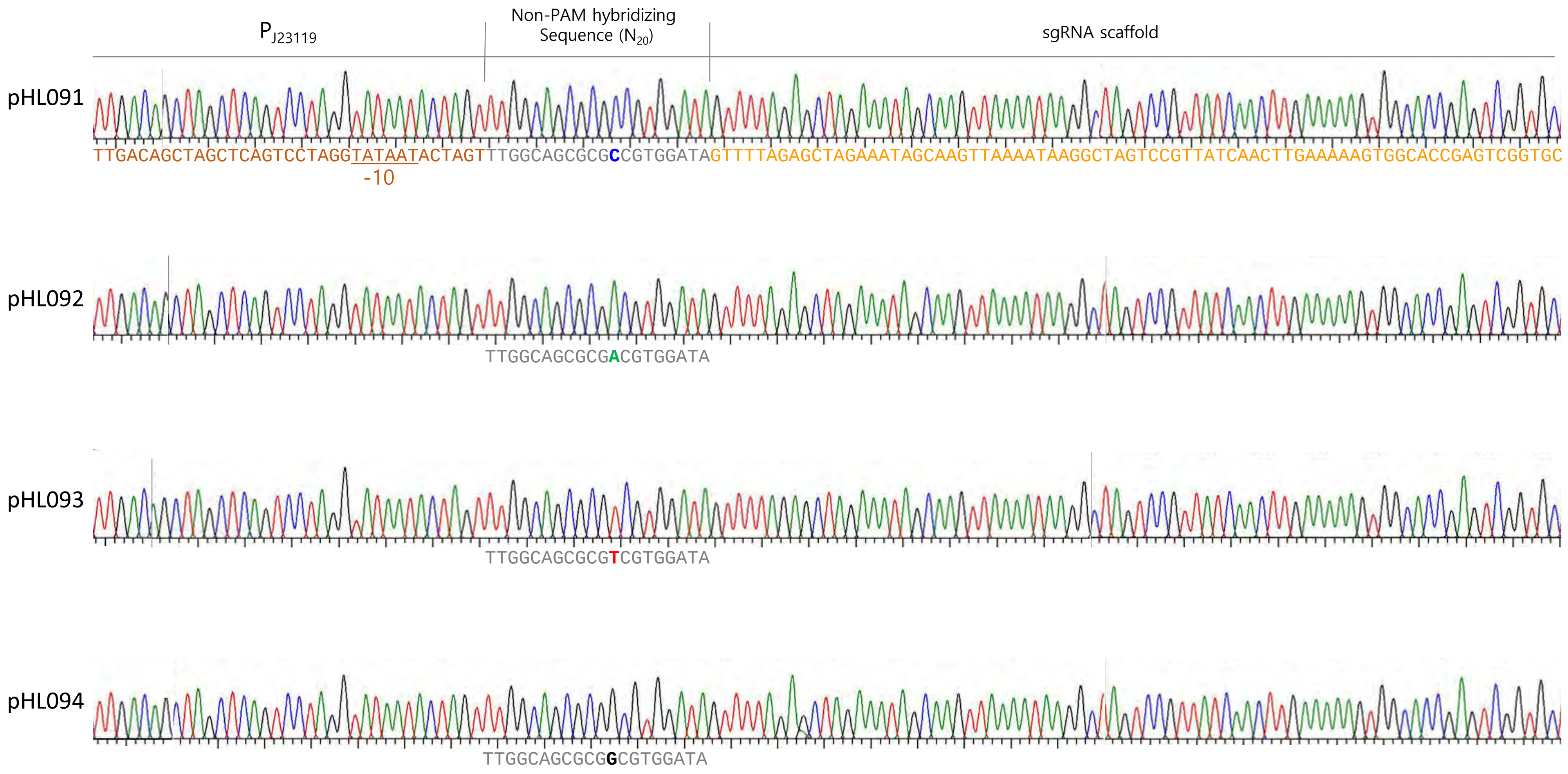

Figure S1. (continued)

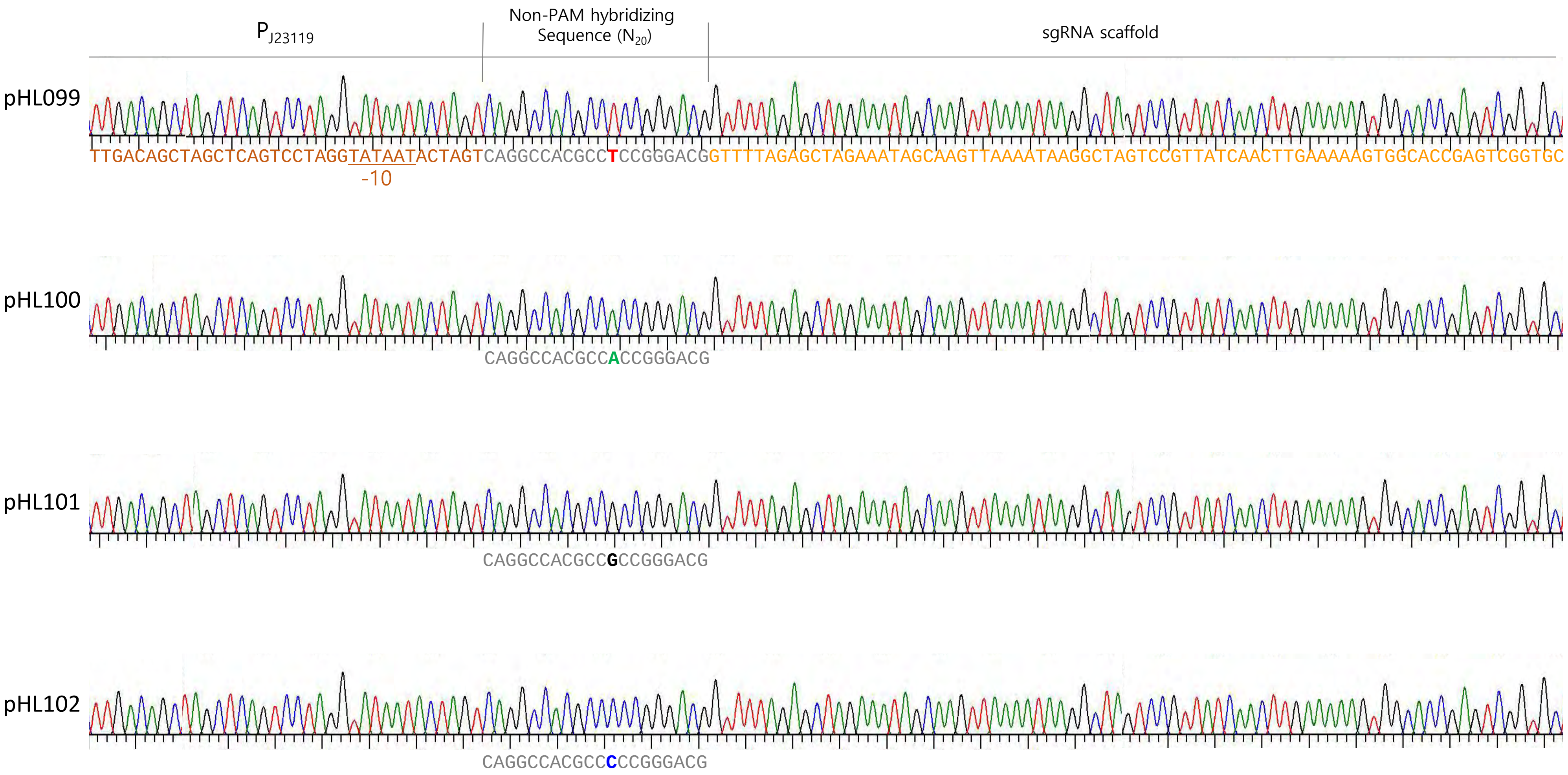

Figure S1. (continued)

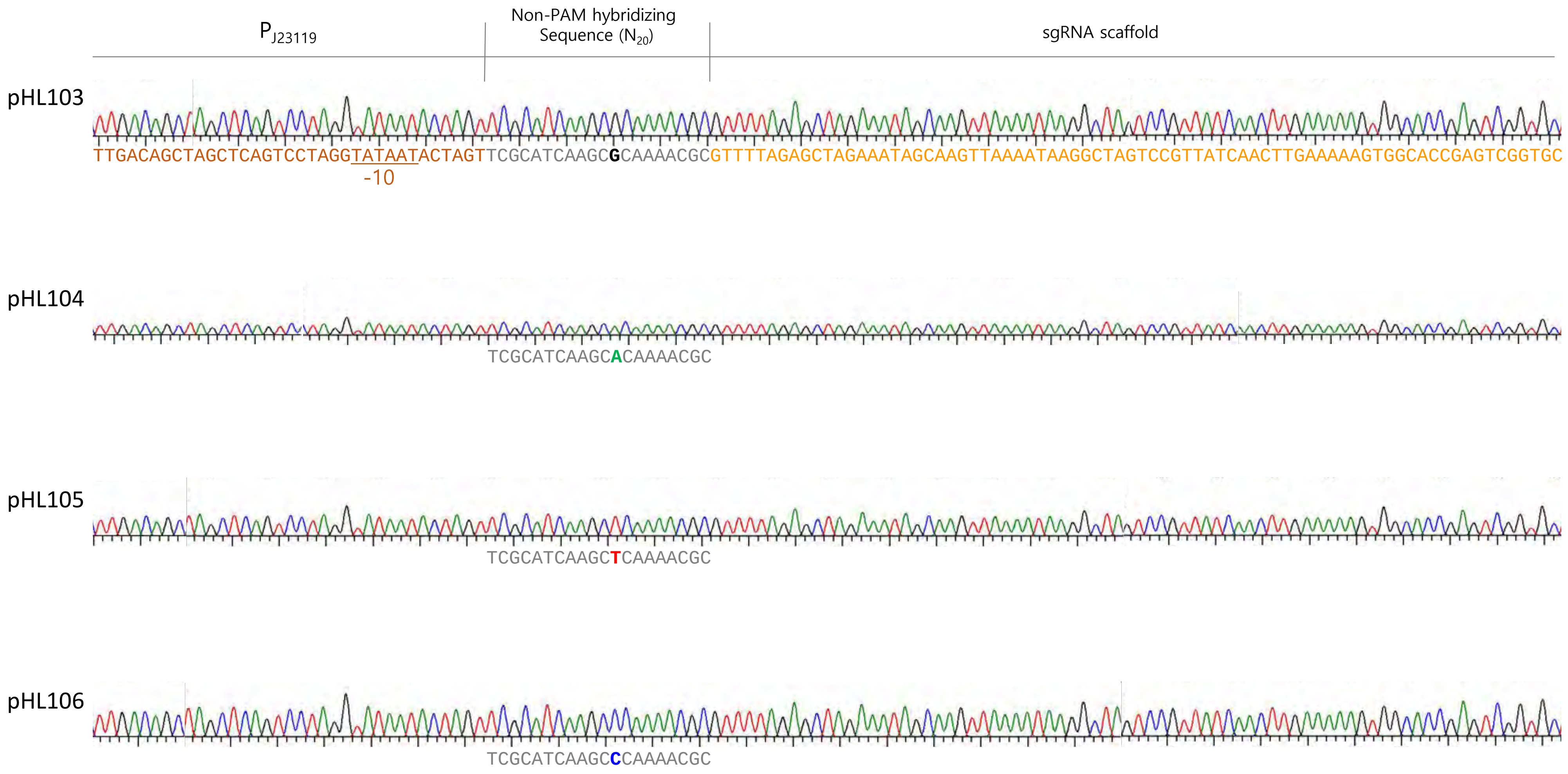

Figure S1. (continued)

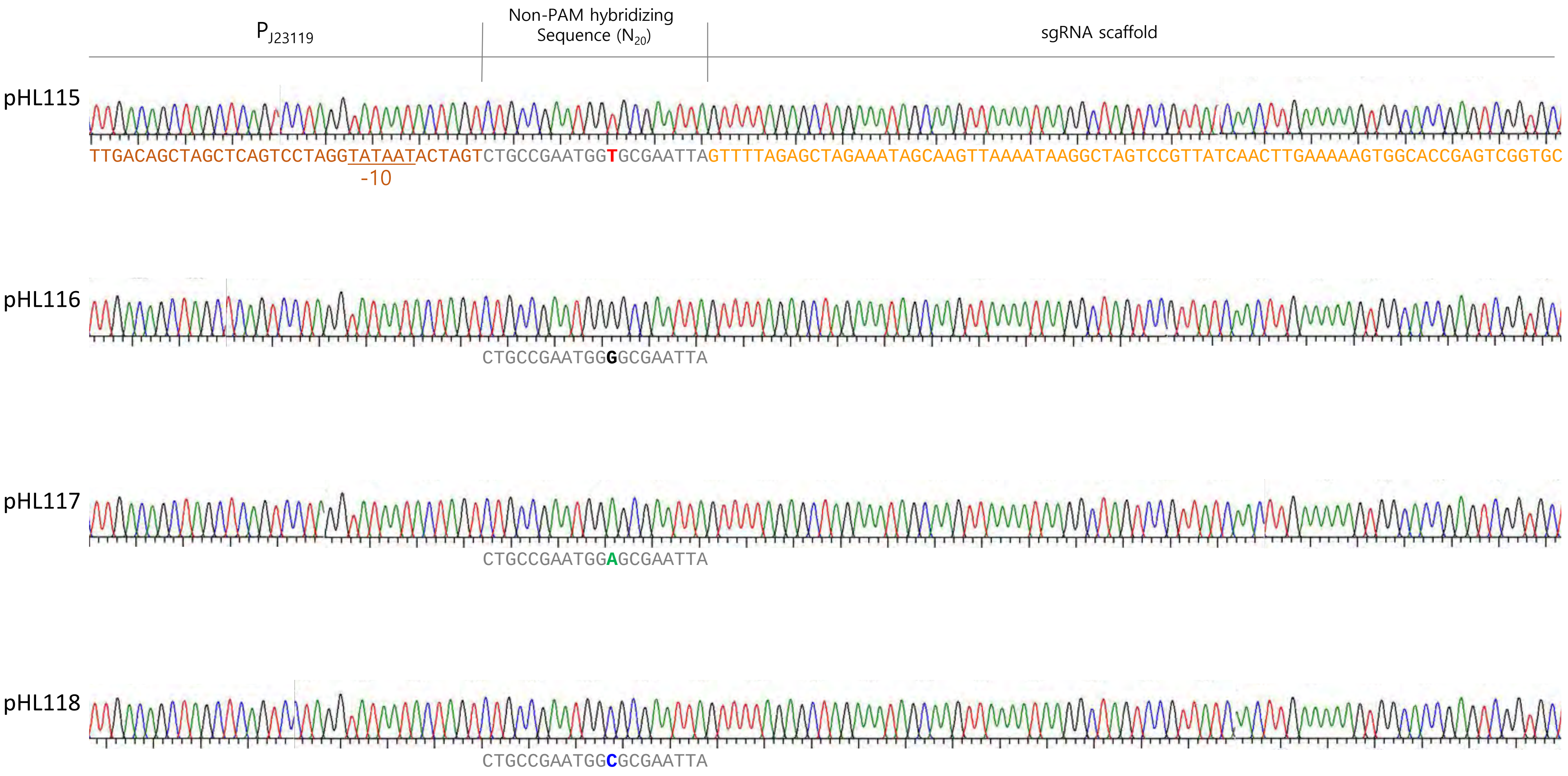

Figure S1. (continued)

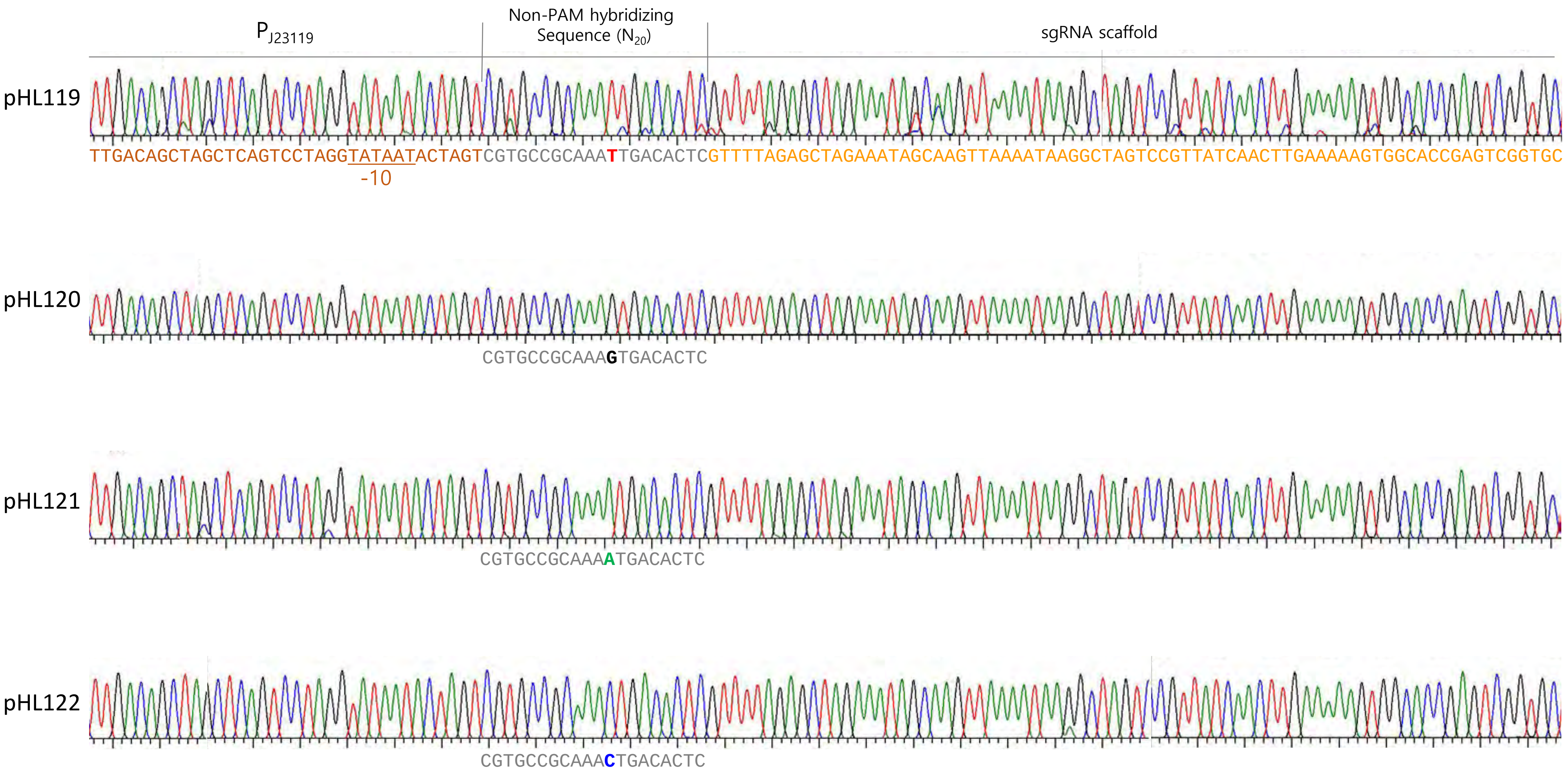

Figure S1. (continued)

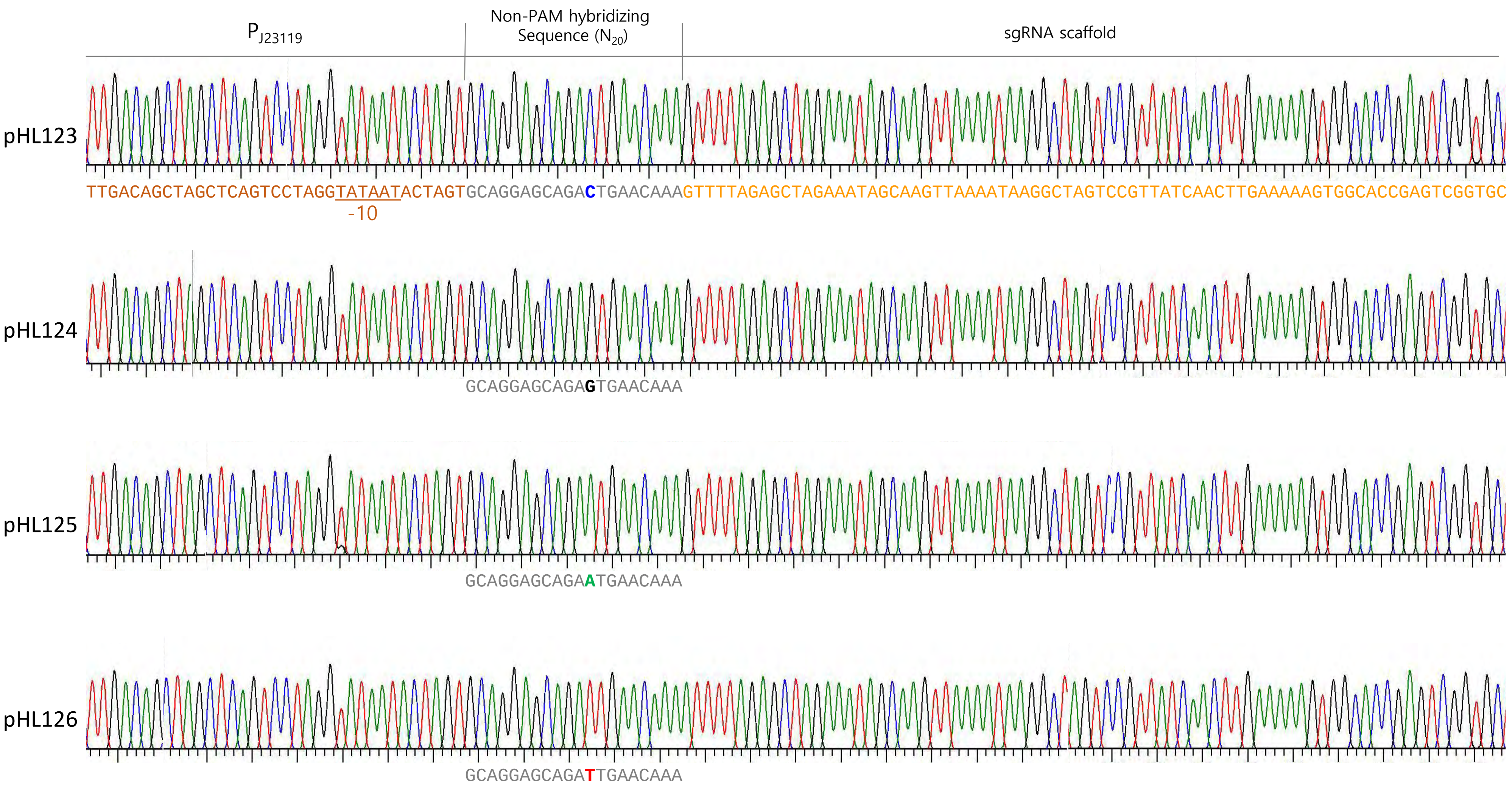

Figure S1. (continued)

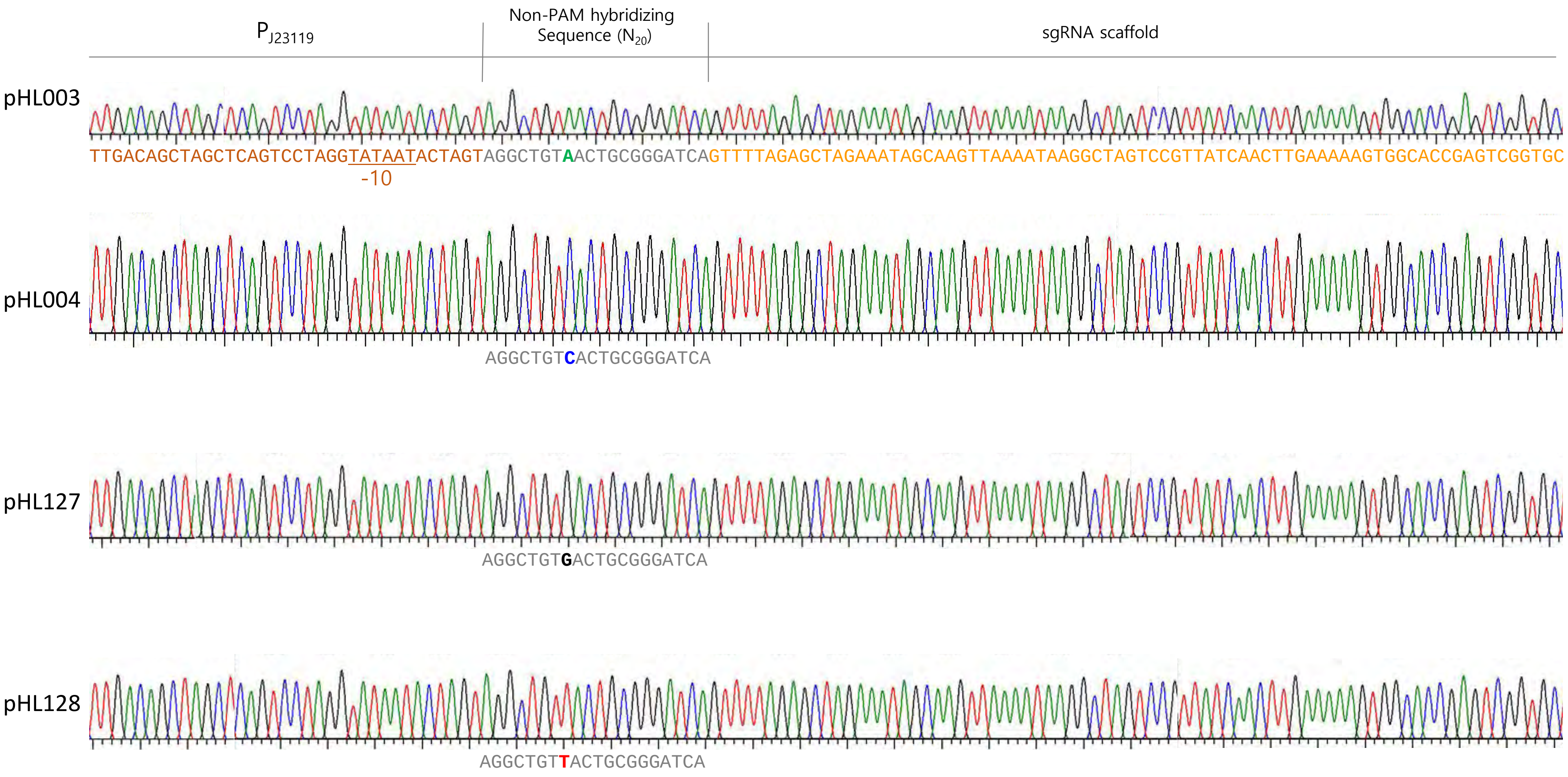

Figure S1. (continued)

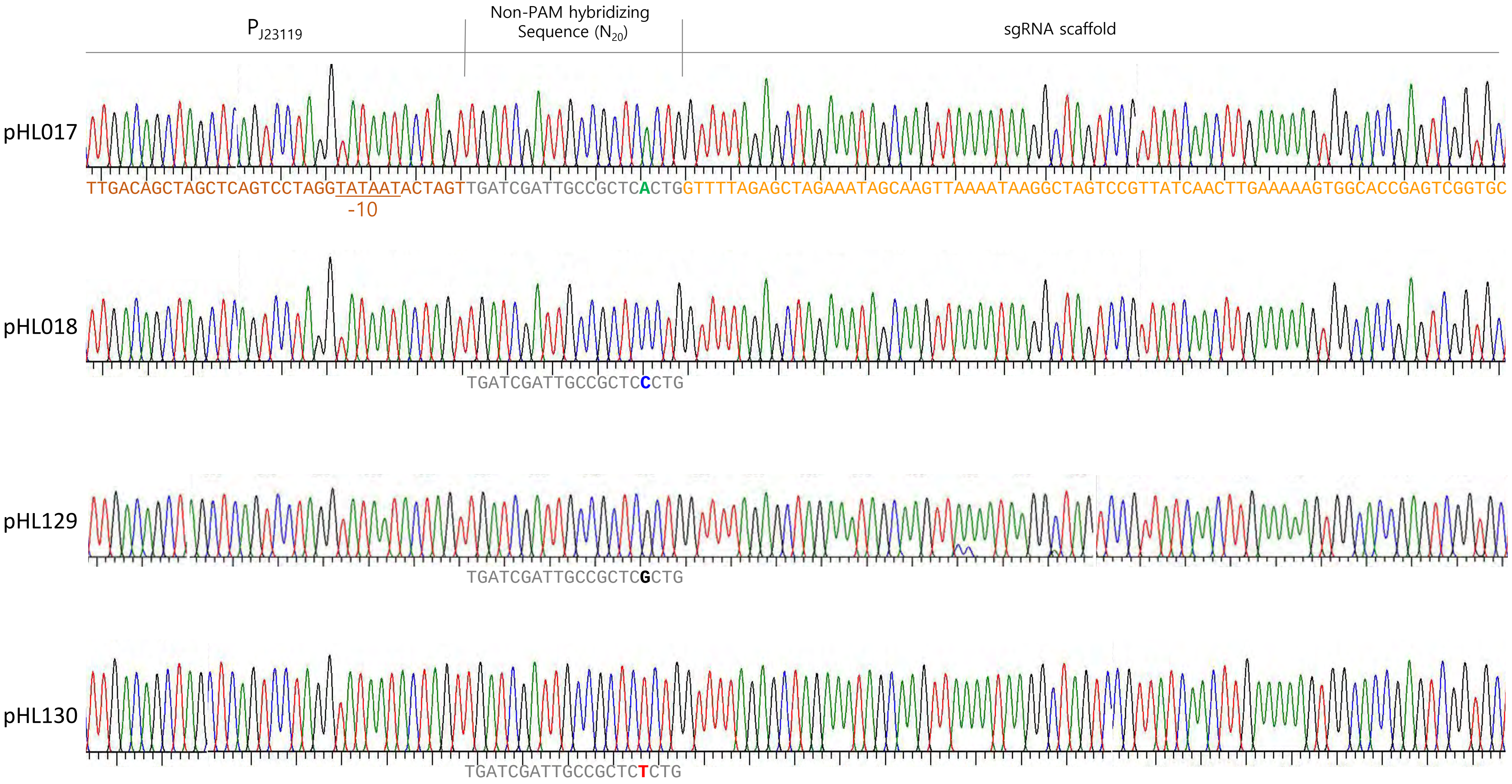

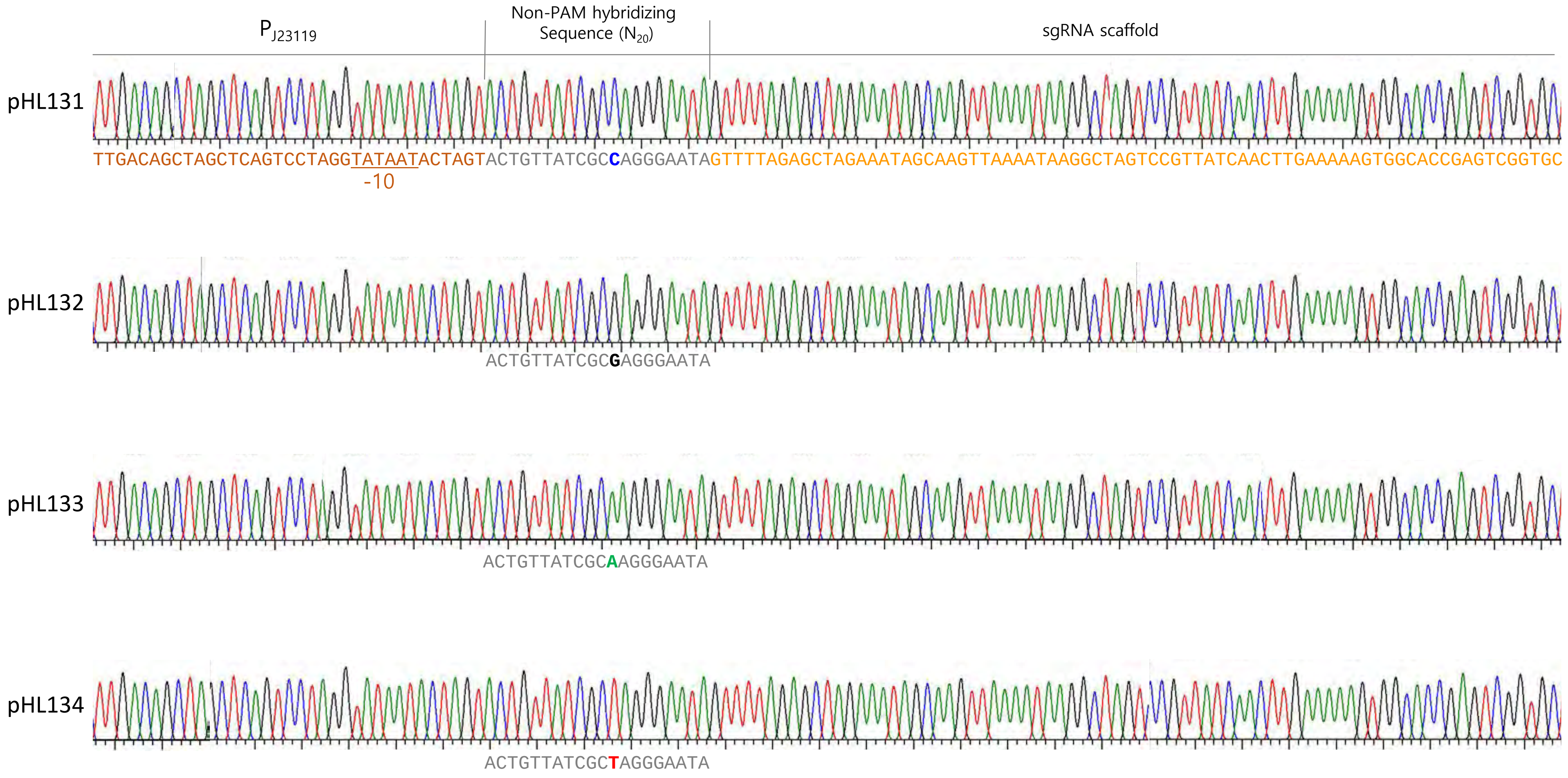

Supplemental Figure S1. Sanger sequences of 64 sgRNA plasmids.
