# Supplementary material for: CRISPR-Cas9-mediated pinpoint microbial genome editing aided by target-mismatched sgRNAs
Source: Genome Res. 2020 May;30(5):768–75. doi: 10.1101/gr.257493.119 (PMC7263196; doi:10.1101/gr.257493.119)
Supplement: Supplemental Material [file supp_gr.257493.119_Supplemental_Fig_S2.pdf]

Figure S2.

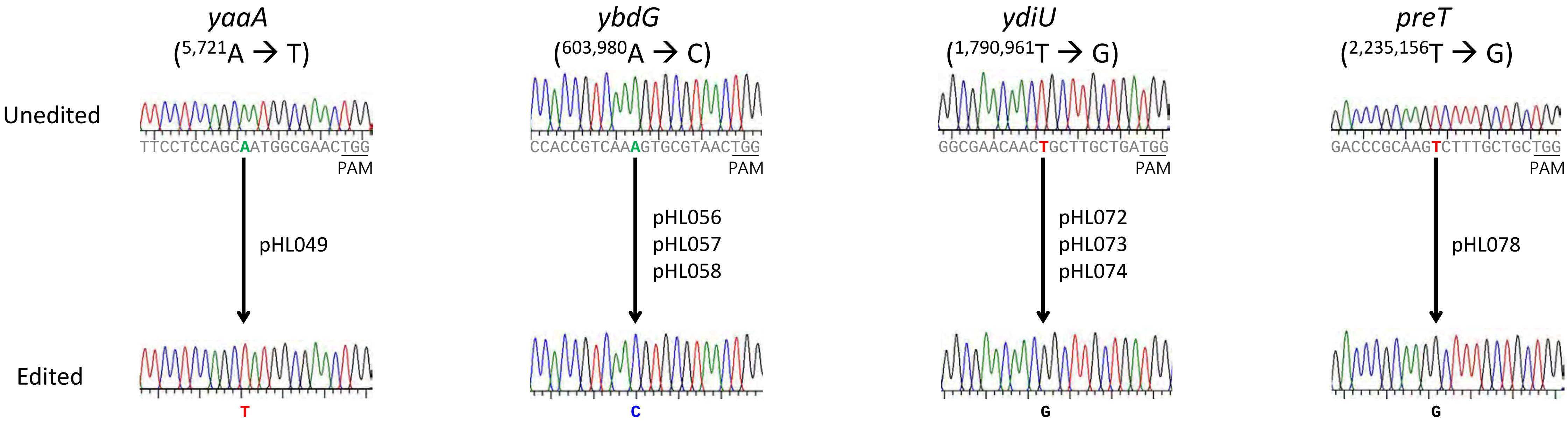

yaaA
(5,721A → T)
ybdG
(603,980A → C)
ydiU
(1,790,961T → G)
preT
(2,235,156T → G)
Unedited
TTCCTCCAGCAATGGCGAACTGG
CCACCGTCAAAGTGCGTAACTGG
GGCGAACAACTGCTTGCTGATGG
GACCCGCAAGTCTTTGCTGCTGG
PAM
PAM
PAM
PAM
pHL049
pHL056
pHL057
pHL058
pHL072
pHL073
pHL074
pHL078
Edited
T
C
G
G

Figure S2. (Continued)

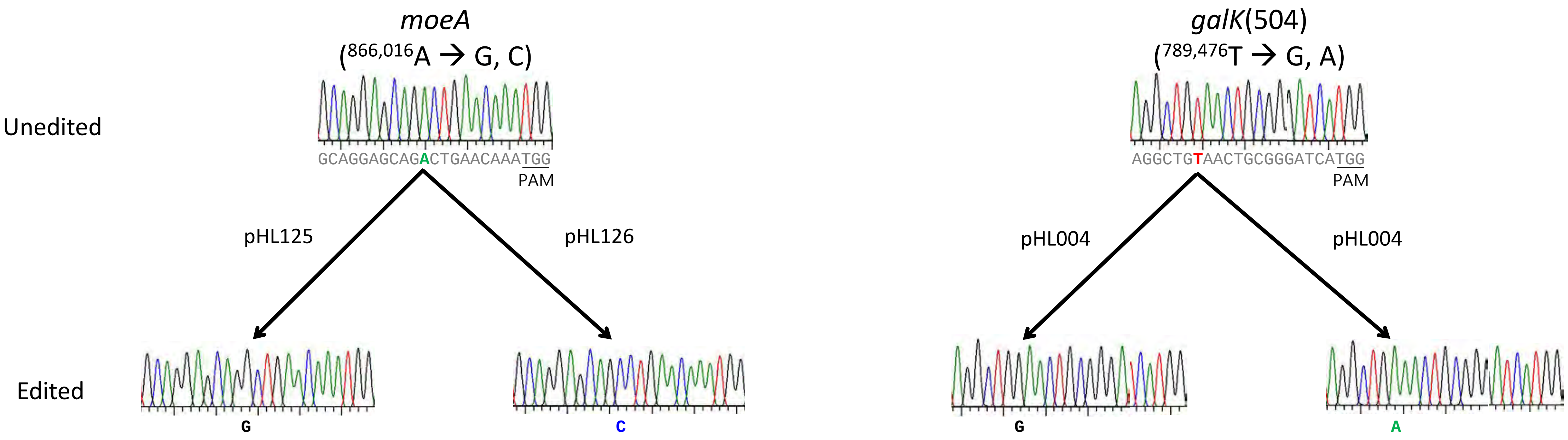

Figure S2. (Continued)

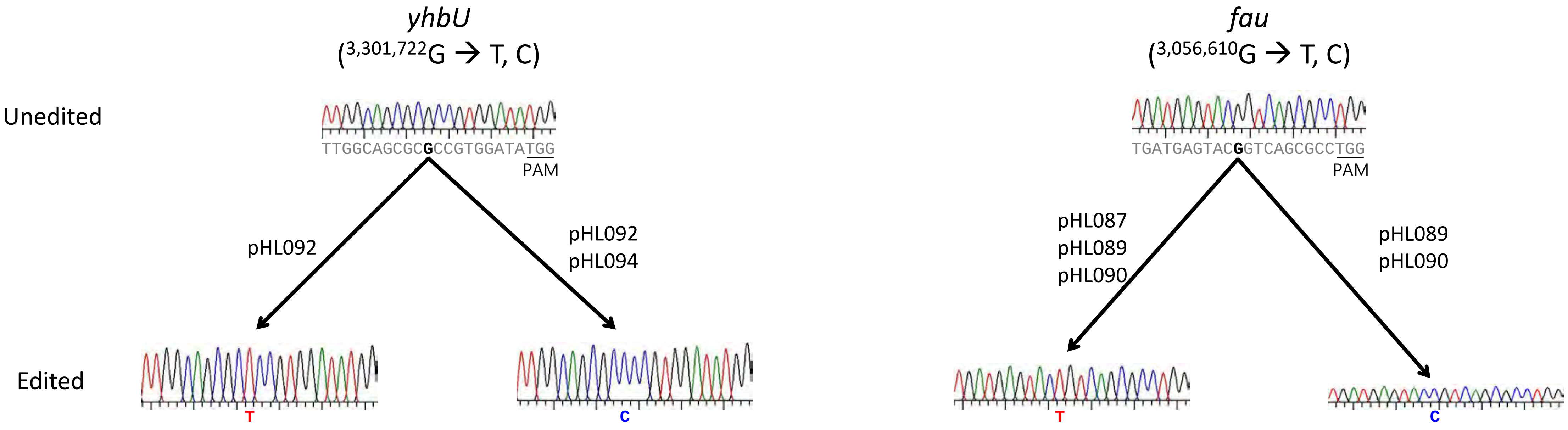

Figure S2. (Continued)

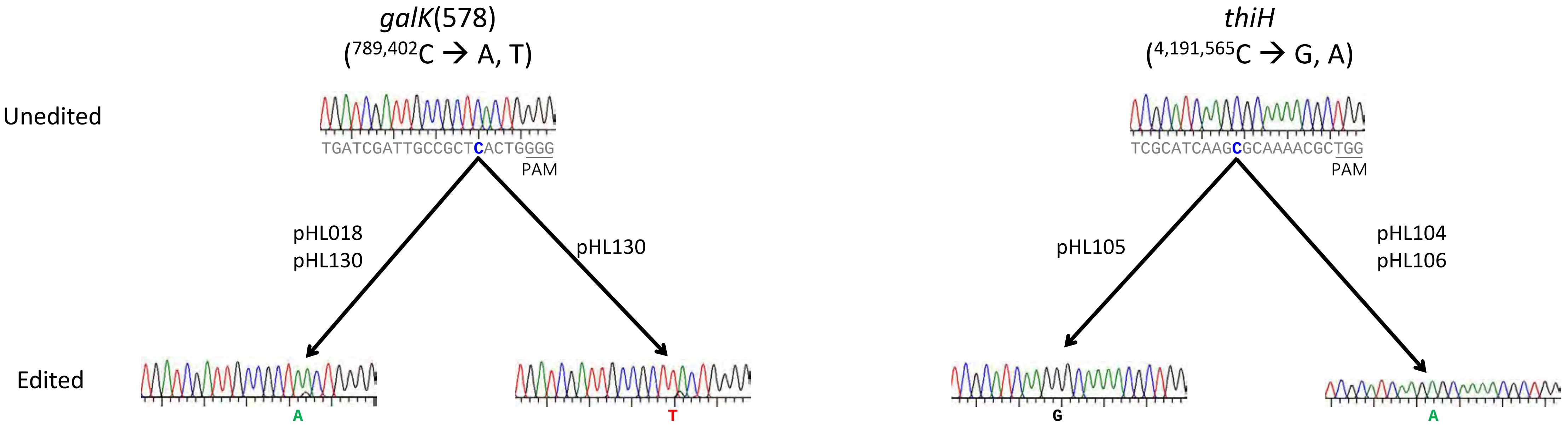

Figure S2. (Continued)

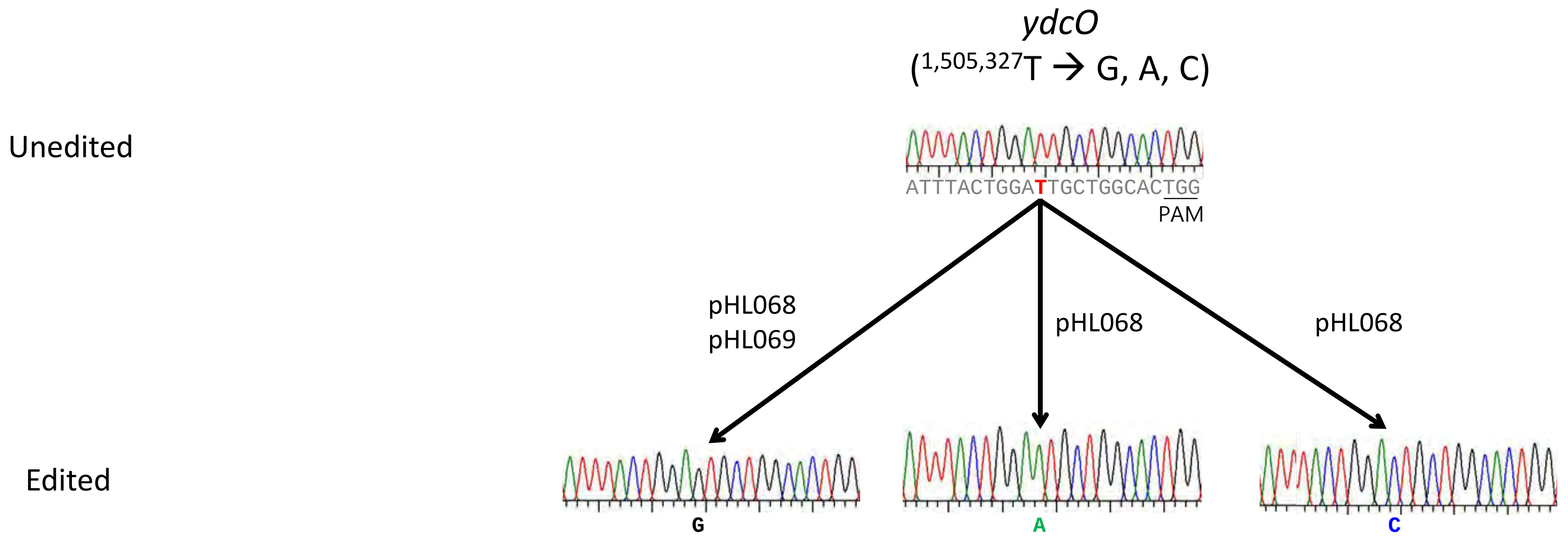

Figure S2. (Continued)

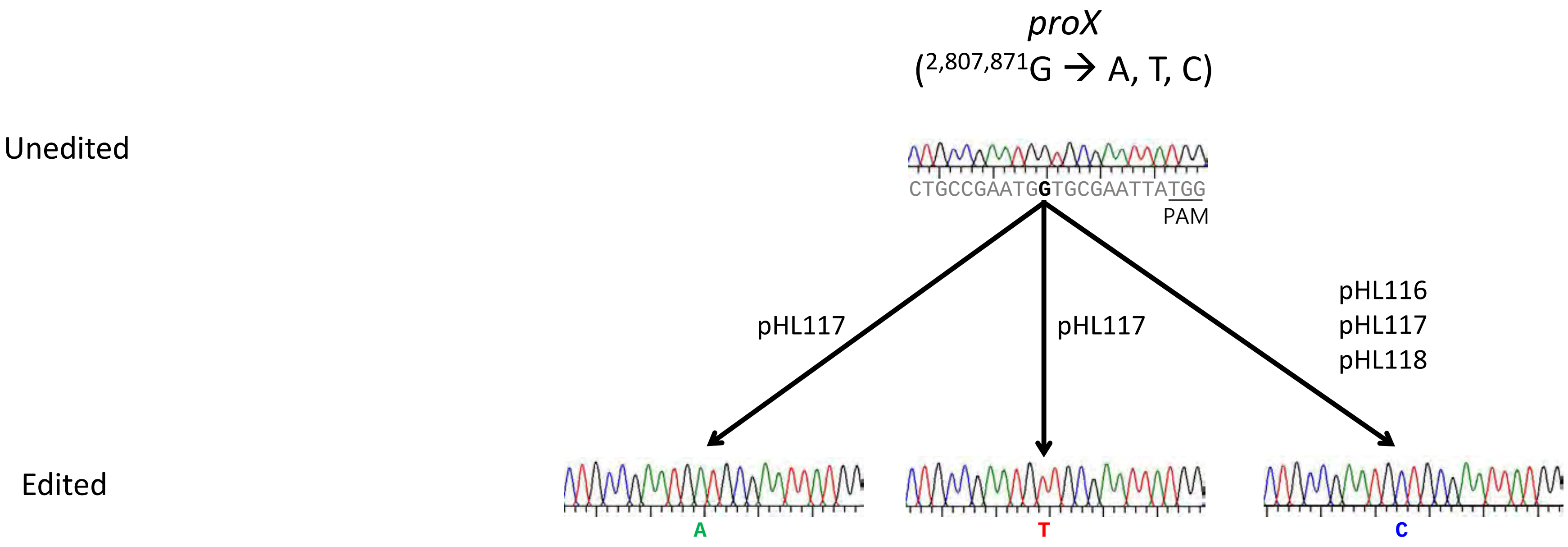

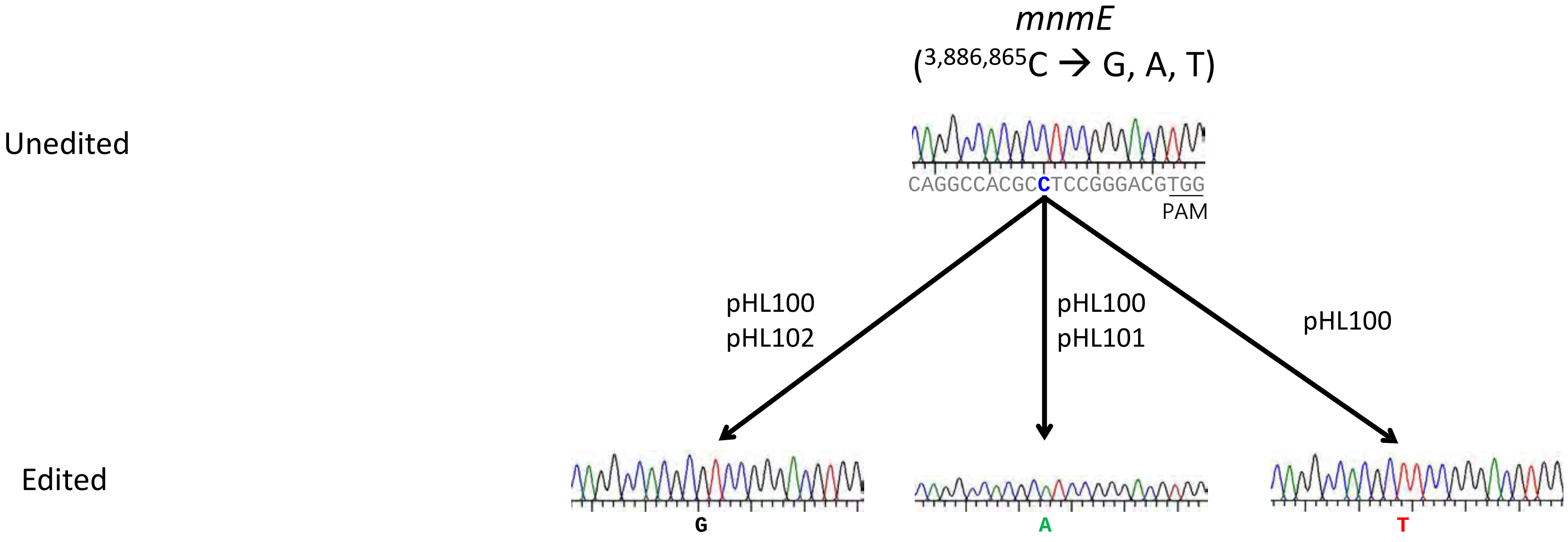

Supplemental Figure S2. Sanger sequences of 25 successful single base editings in the genome.
